# Principles that govern competition or co-existence in Rho-GTPase driven polarization

**DOI:** 10.1371/journal.pcbi.1006095

**Published:** 2018-04-12

**Authors:** Jian-Geng Chiou, Samuel A. Ramirez, Timothy C. Elston, Thomas P. Witelski, David G. Schaeffer, Daniel J. Lew

**Affiliations:** 1 Department of Pharmacology and Cancer Biology, Duke University Medical Center, Durham, North Carolina, United States of America; 2 Department of Pharmacology, University of North Carolina at Chapel Hill, Chapel Hill, North Carolina, United States of America; 3 Department of Mathematics, Duke University, Durham, North Carolina, United States of America; University of California Irvine, UNITED STATES

## Abstract

Rho-GTPases are master regulators of polarity establishment and cell morphology. Positive feedback enables concentration of Rho-GTPases into clusters at the cell cortex, from where they regulate the cytoskeleton. Different cell types reproducibly generate either one (e.g. the front of a migrating cell) or several clusters (e.g. the multiple dendrites of a neuron), but the mechanistic basis for unipolar or multipolar outcomes is unclear. The design principles of Rho-GTPase circuits are captured by two-component reaction-diffusion models based on conserved aspects of Rho-GTPase biochemistry. Some such models display rapid winner-takes-all competition between clusters, yielding a unipolar outcome. Other models allow prolonged co-existence of clusters. We investigate the behavior of a simple class of models and show that while the timescale of competition varies enormously depending on model parameters, a single factor explains a large majority of this variation. The dominant factor concerns the degree to which the maximal active GTPase concentration in a cluster approaches a “saturation point” determined by model parameters. We suggest that both saturation and the effect of saturation on competition reflect fundamental properties of the Rho-GTPase polarity machinery, regardless of the specific feedback mechanism, which predict whether the system will generate unipolar or multipolar outcomes.

## Author summary

Cell morphology is a critical determinant of cell function, and the conserved Rho-family GTPases (Cdc42, Rac, Rho, or Rop in plants) are key regulators of cell morphology. Rho-GTPases self-organize by concentrating into clusters at the cortex, and several mathematical models have been proposed that capture the essential features of such pattern formation. However, it has been unclear how such systems reliably generate either a single cluster (unipolar outcome) or multiple clusters (multipolar outcome). In this paper, we show that a broad class of models for Rho-GTPase polarization all exhibit the ability to switch between a regime in which rapid winner-takes-all competition between clusters yields unipolar outcomes and a regime in which competition is so slow that multipolar outcomes occur at biologically relevant timescales. We find that the switch in model behavior follows a surprisingly simple rule, and elucidate the fundamental principles that underpin that rule. Our theoretical study explains how the same biochemical system can robustly yield unipolar or multipolar outcomes, and makes experimentally testable predictions.

## Introduction

Complex cell morphologies arise, in part, through the specialization of cortical domains (e.g., the apical and basal domains of epithelial cells, or the front and back of migratory cells). Elaboration of such domains involves the local accumulation of active Rho-family GTPases, which regulate cytoskeletal elements to promote specific downstream events, such as vesicle trafficking, membrane deformation, or directed growth [1–3]. For some cells, it is vital to establish a single specialized domain (e.g. the front of a migrating cell), whereas others require the establishment of multiple domains simultaneously (e.g. the dendrites of a neuron) [4, 5]. The mechanistic basis for specifying uni- or multi-polar outcomes remains elusive.

**Fig 1:**
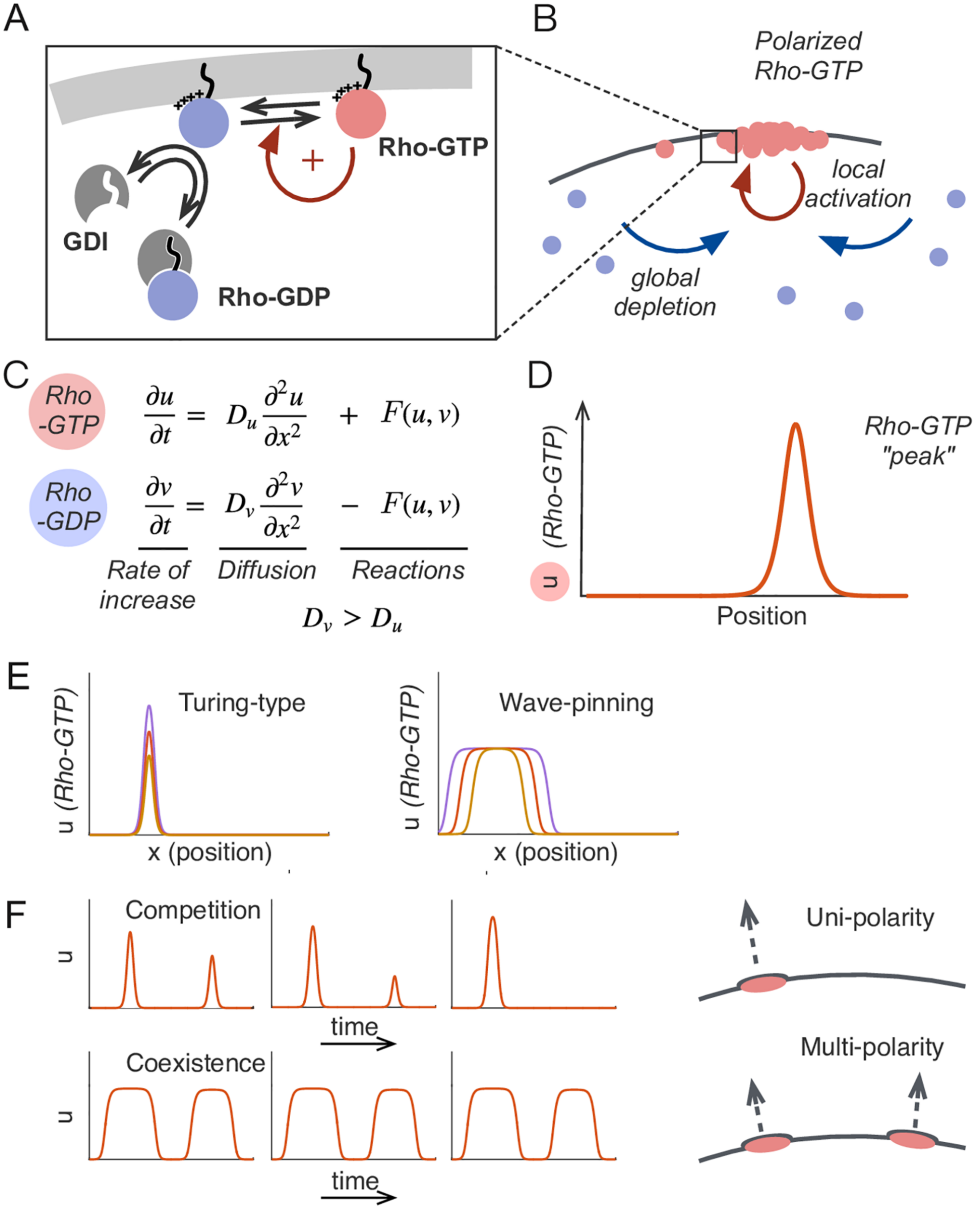
Polarity establishment and competition in mass conserved activator-substrate (MCAS) models. A) Rho-GTPases are tethered to the plasma membrane by prenylation and positive charges. The inactive GDP-bound form, or “substrate”, is preferentially bound by the GDI, masking the prenyl group and the positively charged residues, extracting the substrate to the cytoplasm. The active GTP-bound form, or “activator”, promotes local activation of more substrate, yielding positive feedback. B) Local activation via positive feedback and depletion of the substrate in the cytosol generates an activator-enriched domain on the cortex. C) The interconversions of Rho-GTPases between active and inactive forms can be modeled as a system of two reaction-diffusion equations governing the dynamics of the slowly-diffusing activator *u* and the rapidly-diffusing substrate *v*. The model conserves mass: generation of *u* is precisely matched by consumption of *v* (and vice versa) in the reaction term *F*(*u*, *v*). D) MCAS models generate peaks in the profile of *u*, representing concentrated active Rho-GTPase on the membrane. E) Turing-type models (Eq 4) can generate narrow peaks of different heights, while wave-pinning models (Eq 5) can generate wide mesas of different widths, when total Rho-GTPase content *M* increases. *M* = 4, 6, 10 for Turing-type model and *M* = 30, 40, 50 for wave-pinning model. F) When two peaks of unequal size form in Turing-type models, they compete rapidly and resolve to a single peak, which would lead to unipolar outgrowth (arrow), whereas two mesas of unequal size in Wave-pinning models are meta-stable, which would lead to multi-polar outgrowth (arrow). Parameter values are *a* = 1*μm*^2^, *b* = 1*s*^−1^ and *D*_*u*_ = 0.01*μm*^2^*s*^−1^, *D*_*v*_ = 1*μm*^2^*s*^−1^ for both models, and *k* = 1*μm*^2^ for wave-pinning model. All models were simulated on domain size *L* = 10*μm*.

Rho-family GTPases switch between GTP-bound active and GDP-bound inactive forms (Fig 1A). Active GTPases are tethered to the inner surface of the plasma membrane, where diffusion is slow. In contrast, inactive GTPases are preferentially bound by guanine nucleotide dissociation inhibitors (GDIs), which extract the bound GTPase to the cytoplasm, where their diffusion is comparatively fast. Activated GTPases can promote local activation of cytosolic GTPases via positive feedback. This generates a membrane domain with concentrated active GTPase, concomitantly depleting the cytosolic GTPase pool (Fig 1B). Synthesis and degradation of GTPases occurs on a slow timescale compared to activation and inactivation (for example, in budding yeast the Rho-GTPase Cdc42 polarizes within 2 minutes but has a half-life of more than 20 hours) [6–8]. Thus, the general dynamics of the system can be captured by mass-conserved activator-substrate (MCAS) models, with a slowly-diffusing activator and a rapidly-diffusing substrate (Fig 1C) [9–11]. Such models can generate local peaks of activator, reflecting the establishment of a polarized concentration profile of active GTPase (Fig 1D).

Proposed MCAS models differ primarily in the formulation of the positive feedback mechanism. One set of models yields Turing instability [9, 11], where positive feedback is sufficient to amplify molecular-level fluctuations leading to peak formation. Classically, Turing systems can generate single or multiple peaks [12, 13], depending on whether the size of the modeled domain is larger than a characteristic wavelength dependent on the reaction and diffusion parameters. This has been shown by Linear Stability Analysis (LSA) of the homogeneous steady state (HSS) [14–16]. However, even when multiple peaks emerge from the homogeneous state, most of the peaks in Turing-type MCAS models eventually disappear through a process called “competition”, leaving a single large peak as the winner [11, 17, 18]. Otsuji et al. [11] reasoned that competition arose due to mass-conservation, and further suggested that this might be a general behavior of Turing-type MCAS models. In biological systems, competition-like behavior was observed during polarity establishment in yeast cells, where it was suggested to underlie the growth of only one bud per cell cycle [7, 17, 18].

Another set of models relies on bistable reaction kinetics to produce “wave-pinning” behavior [10, 19–21]. Such models can generate membrane domains with separate phases of uniform high or low activator concentrations connected by a sharp “wavefront”. The wave front spreads laterally but eventually stops (gets pinned) due to depletion of the cytoplasmic substrate, forming stable wide “mesa”-like concentration profiles. In the absence of spatial cues, wave-pinning models can generate multiple mesas when initiated by random fluctuations [10]. Studies done on 1-dimensional wave-pinning model show that multiple mesas appear to be meta-stable [20, 22] and do not readily exhibit competition.

An attractive hypothesis for why some cells are uni-polar and others multi-polar would be that these behaviors arise from differences in the biochemical mechanisms of positive feedback, yielding competition in Turing-type or meta-stability in wave-pinning models. However, some Turing-type MCAS models appear to switch to multi-polarity when domain size [11, 22] or protein amount [17] is increased. Thus, it could be that parameter values (protein concentration, catalytic activity, cell size, etc.) rather than regulatory feedback mechanisms dictate whether uni- and multi- polar outcomes are observed.

Here, we investigate the transient multi-peak scenario, and show that the different models discussed are all capable of generating unipolar or multipolar outcomes. The switch between these outcomes is primarily dictated by a “saturation rule” that is general to MCAS models: Every biologically relevant model in this category has an innate saturation point that sets the maximum local Rho-GTPase concentration. When peaks form such that peak concentrations are well below this saturation point, competition is effective and multi-polar conditions resolve rapidly to a uni-polar steady state. However, if the GTPase concentration in two or more peaks approaches the saturation point, then competition becomes ineffective, and the peaks become meta-stable. Because the saturation rule does not depend on the specifics of the biochemical reactions, our results yield general and testable predictions.

## Results

### MCAS models can produce spatially restricted GTPase enriched domains

Two-species MCAS systems consist of two partial differential equations (PDEs), governing the dynamics of a slowly diffusing activator (GTP-bound GTPase at the membrane) *u*, and a rapidly diffusing substrate (GDP-bound GTPase in the cytoplasm) *v*. In one spatial dimension, these systems take the general form:

$$\begin{array}{c} \frac{\partial u}{\partial t}=D_{u}\frac{\partial^{2}u}{\partial x^{2}}+F\left(u,v\right) \end{array}$$ (1a)

$$\begin{array}{c} \frac{\partial v}{\partial t}=D_{v}\frac{\partial^{2}v}{\partial x^{2}}-F\left(u,v\right) \end{array}$$ (1b)

where the dynamics of *u* and *v* are governed by a diffusion term and a reaction term, *F*(*u*, *v*) (For the dimensionless version, see Supporting Information section 1). To reflect the different compartments (membrane and cytoplasm) populated by the different species, the diffusion constant of *u*, *D*_*u*_, is typically two orders of magnitude smaller than *D*_*v*_, so that *u* spreads much more slowly than *v*. *F*(*u*, *v*) describes the biochemical interconversions between *u* and *v*.

$$\begin{array}{c} F(u,v)=f(u)v-g(u)u \end{array}$$ (2)

For GTPases, the inactive form of the GTPase *v* is converted to the active form *u* through the action of guanine nucleotide exchange factors (GEFs) *f*(*u*), while *u* is converted to *v* through the action of GTPase activating proteins (GAPs) *g*(*u*). The functions *f*(*u*) and *g*(*u*) take into account potential positive feedback mediated by the active GTPase. Because the inactive GTPase is not thought to participate in biochemical reactions other than as a substrate to produce active GTPase, under the assumption of mass action kinetics *v* appears only in the activation term. As the model assumes only the exchange between *u* and *v*, but not synthesis or degradation of either, the system is mass-conserved, so that the total abundance of the GTPase *M* = ∫(*u* + *v*)*dx* is a constant over time.

Generation of a GTPase-enriched domain in MCAS models occurs through positive feedback leading to local accumulation of the activator, *u*, and concomitant depletion of the substrate, *v*. Locally depleted *v* is quickly resupplied from the whole cytoplasm due to its high mobility, resulting in a global depletion of *v*. This reduces the net rate, *F*(*u*, *v*), at which fresh *u* is generated (Eq 2), impeding further growth of the *u*-enriched domain, and the system reaches a steady state. At steady state, reaction and diffusion must be balanced for *u* and *v*:

$$\begin{array}{c} 0=D_{u}\frac{\partial^{2}u}{\partial x^{2}}+F\left(u,v\right) \end{array}$$ (3a)

$$\begin{array}{c} 0=D_{v}\frac{\partial^{2}v}{\partial x^{2}}-F\left(u,v\right) \end{array}$$ (3b)

Given a total protein content *M*, these equations govern the steady state peak shape *u*(*x*) and substrate level *v*(*x*) for a single peak in an MCAS model (Further discussed in Supporting Information section 2).

Positive feedback can occur through *f*(*u*) (i.e. active GTPase locally stimulates GEF activity) or *g*(*u*) (i.e. active GTPase locally inhibits GAP activity). Examples of feedback via GEF activation include the simple Turing-type model *f*(*u*) = *au*^2^, *g*(*u*) = *b*, Goryachev’s simplified model *f*(*u*) = *au*^2^ + *cu*, *g*(*u*) = *b* [9], and Mori’s wave-pinning model $f\left(u\right)=\frac{au^{2}}{1+ku^{2}},g\left(u\right)=b$ [10]. Examples of feedback via GAP inhibition include $f\left(u\right),=1,g\left(u\right)=\frac{b}{{\left(1+u\right)}^{2}}$, which resembles model I in [11]. To illustrate the behaviors of different MCAS models, we simulated examples of Turing-type and wave-pinning MCAS models:

$$\begin{array}{c} F\left(u,v\right)=au^{2}v-bu \end{array}$$ (4)

$$\begin{array}{c} F\left(u,v\right)=\frac{au^{2}}{1+ku^{2}}v-bu \end{array}$$ (5)

With the appropriate choice of parameters, the Turing-type model (Eq 4) yields a peak given any spatial perturbation of the homogeneous steady state, while the wave-pinning model (Eq 5) requires a supra-threshold perturbation to destabilize the homogeneous state. The Turing-type model typically yields a narrow peak at steady state, while the wave-pinning model typically yields a wide mesa (Fig 1E). Simulations with greater total amounts of GTPase *M* yield higher peaks in the Turing-type model, but broader mesas (with the same peak height) in the wave-pinning model (Fig 1E), and simulations initiated with two unequal peaks yield rapid competition in the Turing-type model but apparent co-existence in the wave-pinning model (Fig 1F). These behaviors are all consistent with previous reports [10, 11, 20, 21]; to understand why they yield different outcomes we first revisit the basis for competition.

### Competition between peaks arises from a difference in the ability of unequal peaks to recruit cytoplasmic GTPase

**Fig 2:**
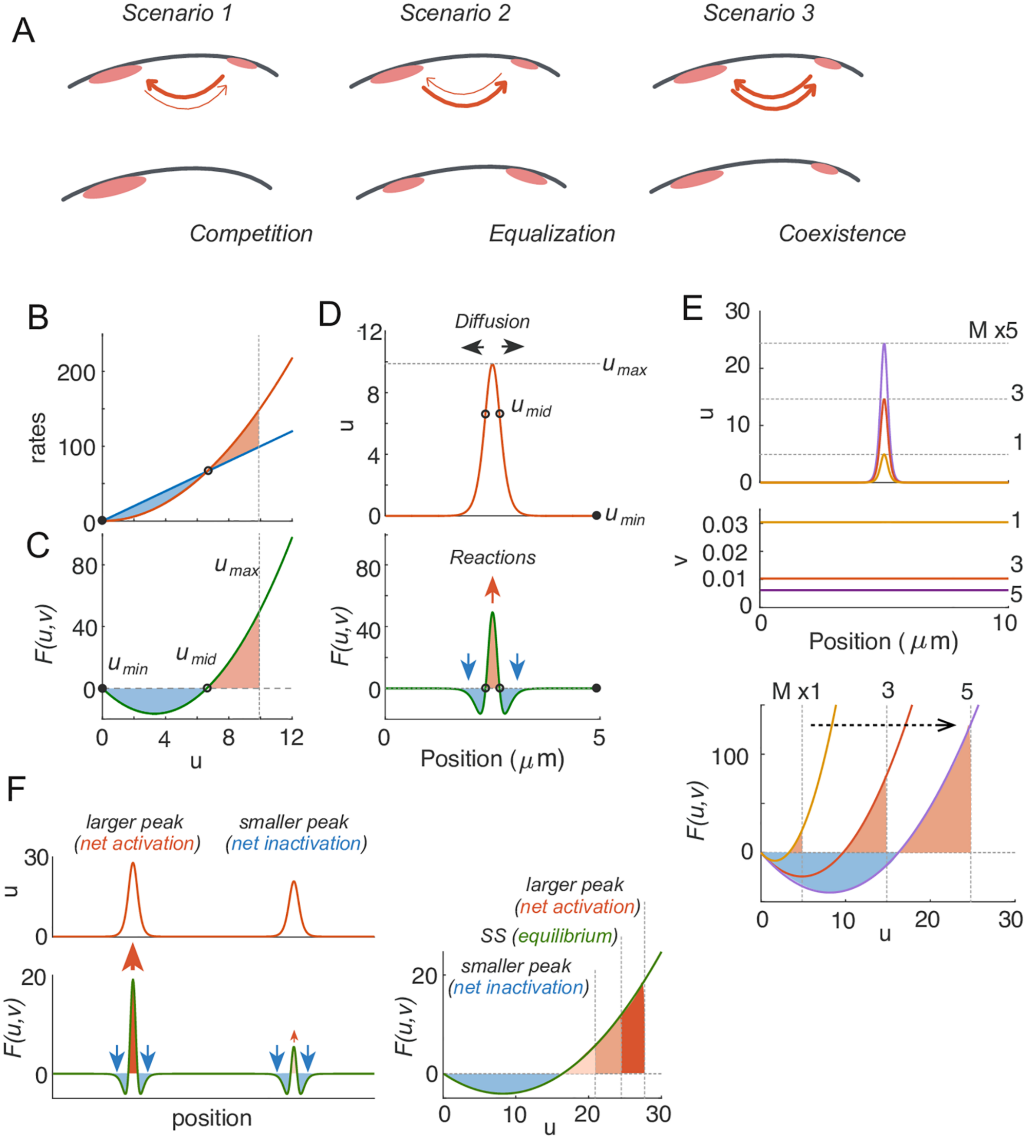
The basis for competition. A) Possible outcomes when there are two unequal clusters of Rho-GTPase in the same cell. Scenario 1: competition occurs if larger clusters recruit GTPase more efficiently than smaller clusters. Scenario 2: equalization occurs if smaller clusters recruit GTPase more efficiently than larger clusters. Scenario 3: co-existence occurs if both clusters recruit GTPase equally well. B-F: Turing-type model with *D*_*v*_ → ∞. B) Rate balance plot: activation and inactivation rates are balanced at two fixed points of *F*(*u*, *v*). Filled circle indicates stable fixed point, and empty circle indicates unstable fixed point. C) Net activation (shaded red) and net inactivation (shaded blue) from the trough (*u*_min_) to the top (*u*_max_) of the peak must be balanced at steady state in 1D. This determines the peak height (*u*_max_). D) Net activation at the center of the peak is balanced by diffusion, which drives GTPase towards the flanks, where there is net inactivation. The activation curve and net reaction curves were plotted given *v* at steady state in B, C, and D. E) If total GTPase content *M* is raised, the model generates higher peaks (larger *u*_max_), accompanied by more severely depleted *v*, which lowers *F*(*u*, *v*) such that the blue and red shaded areas are once again balanced. F) When two peaks are present, they share the same *v* and hence the same *F*(*u*, *v*) curve. The larger peak will always have excess net activation, and the smaller peak will always have excess net inactivation, so competition is inevitable. Parameter values used: *a* = 1*μm*^2^, *b* = 1*s*^−1^ and *D*_*u*_ = 0.01*μm*^2^*s*^−1^, *D*_*v*_ = ∞. All models were simulated on domain size *L* = 10*μm*.

When two unequal peaks are present in the same domain, each peak recruits GTPase from the cytoplasm, thereby globally depleting cytoplasmic GTPase until cytoplasmic concentration reaches a quasi-steady state. As exchange of GTPase between each peak and the cytoplasm is dynamic, the two peaks are now effectively recruiting GTPase from one another. If the larger peak (the one that contains more GTPase) recruits GTPase more effectively, it will grow at the expense of the smaller peak, eventually yielding a uni-polar outcome (Fig 2A, scenario 1). If instead, the smaller peak recruits GTPase more effectively, then it will grow while the larger peak shrinks, eventually yielding two equal peaks, as observed in some more complex models [17] (Fig 2A, scenario 2). If two unequal peaks recruit GTPase equally, then the two unequal peaks would simply coexist (Fig 2A, scenario 3).

To understand how these considerations play out for different peaks, we need to know whether the larger peak recruits more GTPase. To assess how much GTPase would be recruited to a specific peak, consider first the Turing-type model (Eq 4) in the limit *D*_*v*_ → ∞. This model combines a quadratic (in *u*) activation term with a linear inactivation term (Fig 2B). Thus, for a fixed value of *v*, there are two values of *u* at which activation and inactivation balance each other precisely (i.e. fixed points of the net reaction curve *F*(*u*, *v*) in Fig 2C, denoted as *u*_min_ and *u*_mid_). Given the concentration profile of a peak (Fig 2D, upper panel), *F*(*u*, *v*) determines whether any given location on the membrane will gain GTPase from the cytoplasm or lose GTPase to the cytoplasm (Fig 2D, lower panel). At the trough in Fig 2D (*u*_min_), *u* approaches the lower fixed point of *F*(*u*, *v*), yielding no net gain or loss of GTPase. On the lower flanks of the peak, *u* values lie between *u*_min_ and *u*_mid_, and inactivation outpaces activation, so there is a net loss of *u* (Fig 2B, 2C and 2D). When *u* rises above *u*_mid_, up until the top of the peak (*u*_max_), there is net recruitment of GTPase from the cytoplasm (Fig 2B, 2C and 2D). At steady state, diffusion from the center of the peak to the flanks balances these flows of GTPase, requiring a narrow peak (where negative $\frac{\partial^{2}u}{\partial x^{2}}$ counteracts net recruitment at the center: Eq 3) (Fig 2D).

At steady state, the net loss from the region between *u*_min_ and *u*_mid_ (blue area in Fig 2B and 2C) must be balanced by the net recruitment from the region between *u*_mid_ and *u*_max_ (red area in Fig 2B and 2C) (For analytical support of this argument, see Supporting Information section 2. This is also referred to as the wave-pinning condition in [10]). If we started from a steady state peak and increased *M*, *u*_max_ would increase and the red area would become larger. To reach a steady state, *v* would have to decrease, weakening the influence of the activation term in *F*(*u*, *v*) (Fig 2E), and equalizing the red and the blue areas at steady state (though each area would end up larger than for the initial peak). Another way to understand this is the following argument: If we added more inactive GTPase to the depleted cytoplasm beneath an existing steady state peak, the activation rate (linear in *v*) would increase, causing the peak to grow and depleting inactive GTPase. When *v* gets back down to its starting steady state level, the peak will be larger, so both the activation and inactivation rates will be larger. However, the activation rate will dominate due to the non-linear positive feedback, causing further depletion of *v* until the net rates balance. Thus, for any *F*(*u*, *v*) that encodes a non-linear positive feedback, a larger peak will recruit cytoplasmic GTPase more strongly and cause a more severe cytoplasmic depletion.

Now consider a scenario in which two unequal peaks are present in the same domain. Both peaks would grow until cytoplasmic *v* becomes sufficiently depleted. In the limit where *D*_*v*_ → ∞, *v* is spatially homogeneous, so the same net reaction applies to both peaks, but the peaks will have a different *u*_max_ (Fig 2F). The overall recruitment or loss of GTPase for each peak *u*(*x*) is given by:

$$\begin{array}{c} \int F(u,v)dx \end{array}$$ (6)

The more GTPase there is in the larger peak compared with the smaller one, the larger the difference in “recruitment power” between them (Fig 2F). Thus, in a scenario with unequal peaks in the same domain, the larger peak experiences a net gain of GTPase, while the smaller peak experiences a net loss, further exacerbating the inequality between the two peaks until the smaller peak is eliminated. The Turing model (Eq 4) with *D*_*v*_ → ∞ always competes to yield a uni-polar endpoint (scenario 1 in Fig 2A).

The argument above requires only mass-conservation and non-linear positive feedback, which is a core requirement for polarization in general [12]. Therefore, it would seem that all MCAS models should compete, regardless of the specific *F*(*u*, *v*). To verify this, we generated steady states with two symmetric peaks in a domain, and performed linear stability analysis to show that such steady states are unstable (Supporting Information section 3). Perturbations that destabilize the steady state yield either competition between the peaks or merging of the peaks. Here we focus on competition. Our analysis in the limit of *D*_*v*_ → ∞ indicates that given sufficient time, two peaks will always compete to produce a single peak. This result does not depend on the form of *F*(*u*, *v*).

### Competition slows down dramatically due to saturation

**Fig 3:**
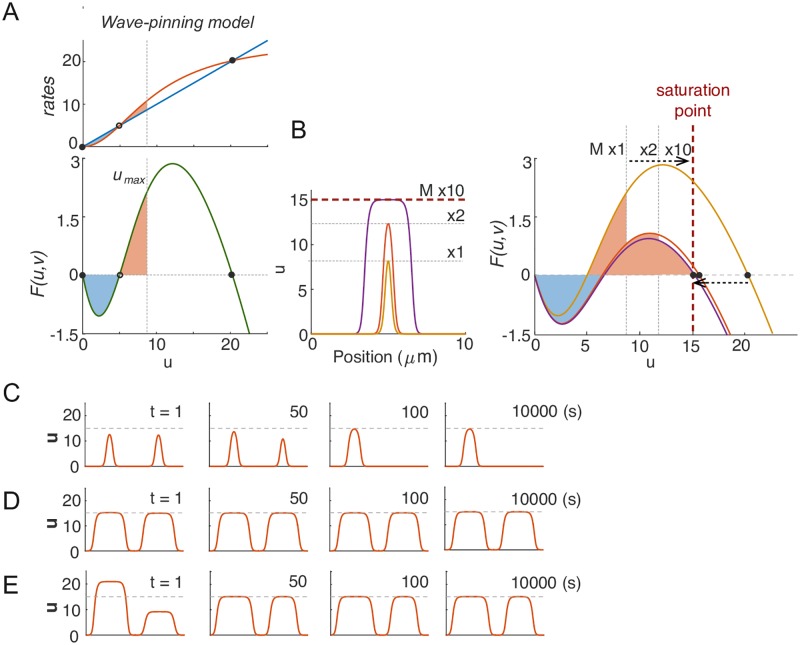
Wave-pinning models can generate coexisting clusters due to saturation. A) The wave-pinning model has a saturable activation term, introducing a third fixed point in *F*(*u*, *v*). Dashed line indicates *u*_sat_. Circles indicate stable (filled) and unstable (empty) fixed points. B) As total GTPase levels *M* increase, the peaks get higher until *u*_max_ reaches the saturation point (the third fixed point), after which peaks broaden into mesas. C) With *M* = 40, two identical peaks were perturbed by 1% at *t* = 0*s*. The resulting competition led to a single-peak steady state within 100*s*. D) With *M* = 200, the same 1% perturbation did not result in noticeable competition in 10000*s*. E) Starting from the same two-peak steady state as in D, we introduced a large 50% perturbation. The two mesas quickly evolved back to the original *u*_max_, and then persisted for 10000*s*. *k* = 0.01*μm*^2^. Other parameters same as Fig 2.

If competition (scenario 1 in Fig 2A) applies to all MCAS models, then why did we not observe competition in simulations of the Wave-pinning model (Fig 1G)? In contrast to the Turing-type model (Eq 4), the reaction term of the Wave-pinning model (Eq 5) has saturable positive feedback, introducing a third fixed point in *F*(*u*, *v*) (Fig 3A). When the total protein content in the system is small, *u*_max_ does not approach this fixed point (Fig 3B). Under these conditions, narrow peaks compete with each other to yield a uni-polar outcome, as with the Turing-type model (Fig 3C). But when protein content of the peak is increased, *u*_max_ approaches the third fixed point, and the reaction rate *F*(*u*, *v*) at the top of the peak approaches zero (Fig 3B). To satisfy the steady-state condition (Eq 3a), $\frac{\partial^{2}u}{\partial x^{2}}$ must also approach zero. In other words, the top of the peak must broaden to become a wide mesa. Once this occurs, increasing *M* only negligibly increases *u*_max_, and instead of developing higher peaks the model develops broader mesas with comparable *u*_max_ (Fig 3B). As *u*_max_ saturates in these peaks, we shall call this maximum value the “saturation point” (*u*_sat_) of the model.

When *u*_max_ approached the saturation point *u*_sat_, simulations with two saturated mesas did not show obvious competition (Fig 3D). Applying a drastic perturbation in which 50% of the GTPase in one mesa was transferred to the other led to a rapid adjustment with both mesas returning to an almost identical *u*_max_ but with different widths, after which the unequal mesas co-existed for prolonged simulation times (Fig 3E) (Note that the two peaks did not “equalize”: they retained unequal total GTPase content.) Thus, the same model can yield rapid competition or competition so slow as to yield prolonged co-existence, simply as a result of varying the total amount of GTPase in the system.

**Fig 4:**
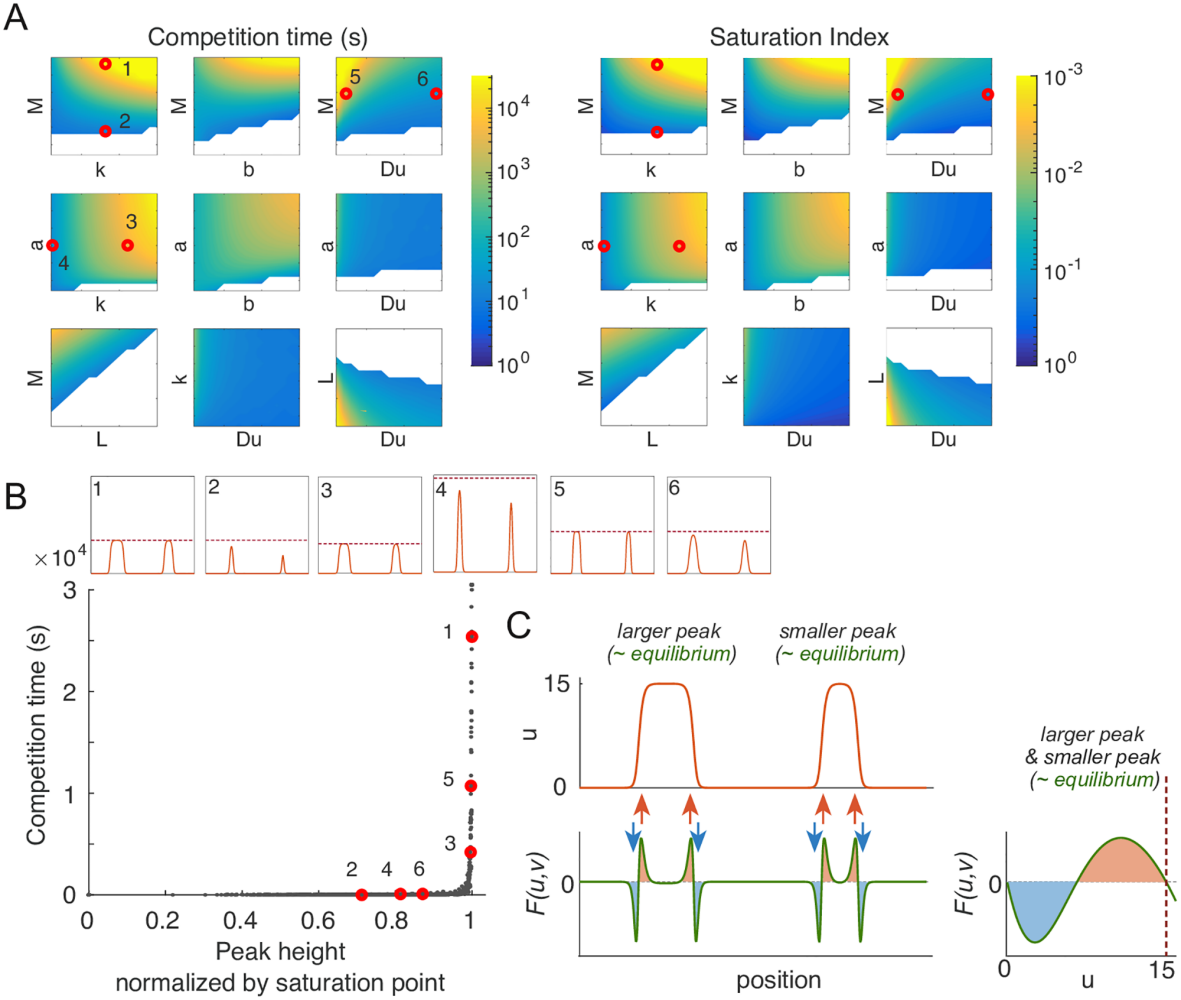
Saturation is a major contributor to differences in competition times. A) Competition time and saturation are tightly correlated. Competition time (*s*) is shown in color (note log scale). Saturation index is defined here as (*u*_sat_ − *u*_max_)/*u*_sat_, and colored in inverse log scale (smaller saturation index indicates peaks are closer to saturation). Basal parameters: *a* = 1*μm*^2^*s*^−1^, *b* = 1*s*^−1^, *k* = 0.01*μm*^2^ and *D*_*u*_ = 0.01*μm*^2^*s*^−1^, *D*_*v*_ = ∞, *M* = 40, *L* = 20*μm*. Each color plot represents a 15-fold parameter variation from 0.2× to 3× of the basal parameter value. White regions indicate parts of parameter space where polarized states collapse to homogeneous states. Numbered red dots correspond to the simulations illustrated in the inset of panel B). B) Each of the simulations performed for panel A) is plotted as one dot. Competition time (vertical axis) is plotted against peak height *u*_max_ normalized to the saturation point *u*_sat_ for that simulation (horizontal axis). Inset graphs indicate starting conditions for the selected simulations with parameters indicated by red dots in A). C) When two mesas coexist, they share the same *F*(*u*, *v*) curve and almost the same *u*_max_. Thus, the wider peak has a negligible recruitment advantage over the narrower one.

To investigate more broadly how model parameters might influence the timescale of competition between peaks, we simulated competition between two unequal peaks in the Wave-pinning model, in the limit with *D*_*v*_ → ∞. If we start with a two-peak steady state and noise, the two peaks will eventually resolve to one, given sufficient time. As a measure of competition time that should be insensitive to the precise degree of the noise, we tracked the time it took for unequal peaks with active GTPase content ratio 3:2 to evolve to a content ratio of 99:1. Parameter changes caused dramatic changes in competition times, color coded on a log scale in Fig 4A. Notably, increasing *M* always led to slower competition (Fig 4A, left panel). As discussed above, increasing GTPase content causes *u*_max_ to approach the saturation point. Defining a saturation index in terms of how closely *u*_max_ at the two-peak steady state approached the saturation point ((*u*_sat_ − *u*_max_)/*u*_sat_), we found that the effects of varying parameters on the saturation index closely paralleled the parameter effects on the timescale of competition (Fig 4A, right panel). A similar congruence was observed using peak width as a different measure of how closely the system approaches saturation (Fig S5B in S1 Text). These findings suggest that a large majority of the variation in competition times can be explained simply by the degree to which peaks in the model approach the saturation point.

If we plot competition time against *u*_max_ normalized to the saturation point, all of the simulations with different parameter values display one of two clearly distinct behaviors (Fig 4B). Parameter changes can alter GTPase content in the peaks (Fig 4A and 4B, point 1 vs 2), the saturation point (point 3 vs 4), or the shapes of the peaks (point 5 vs 6). In all cases, whenever *u*_max_ is not close to saturation, competition occurs rapidly. Conversely, as *u*_max_ approaches the saturation point, competition slows sharply and the two-peak situation becomes meta-stable, resembling the co-existence scenario 3 in Fig 2A.

The basis for the drastically slowed competition in simulations with peaks close to saturation can be intuitively understood in terms of each peak’s “recruitment power” (Eq 6). When peaks approach saturation, unequal peaks differ in width but have almost identical *u*_max_ and hence only a negligible difference in recruitment power (Fig 4C). In the saturated regions of peaks, *F* = 0, so these areas do not directly contribute to overall recruitment. For that reason, the extra GTPase in a broader peak does not give it a significant advantage over the narrower peak, and the driving force for competition is negligible.

Analysis of the eigenvalues from linear stability analysis of this system shows that the timescale of competition slows exponentially as the peaks increase in width by saturation. This conclusion, again, is general to all MCAS models and can be applied to all formulations *F*(*u*, *v*) that allow a third fixed point (Supporting Information section 4, Fig. S5A).

### Local cytoplasmic depletion also leads to saturation and slow competition

**Fig 5:**
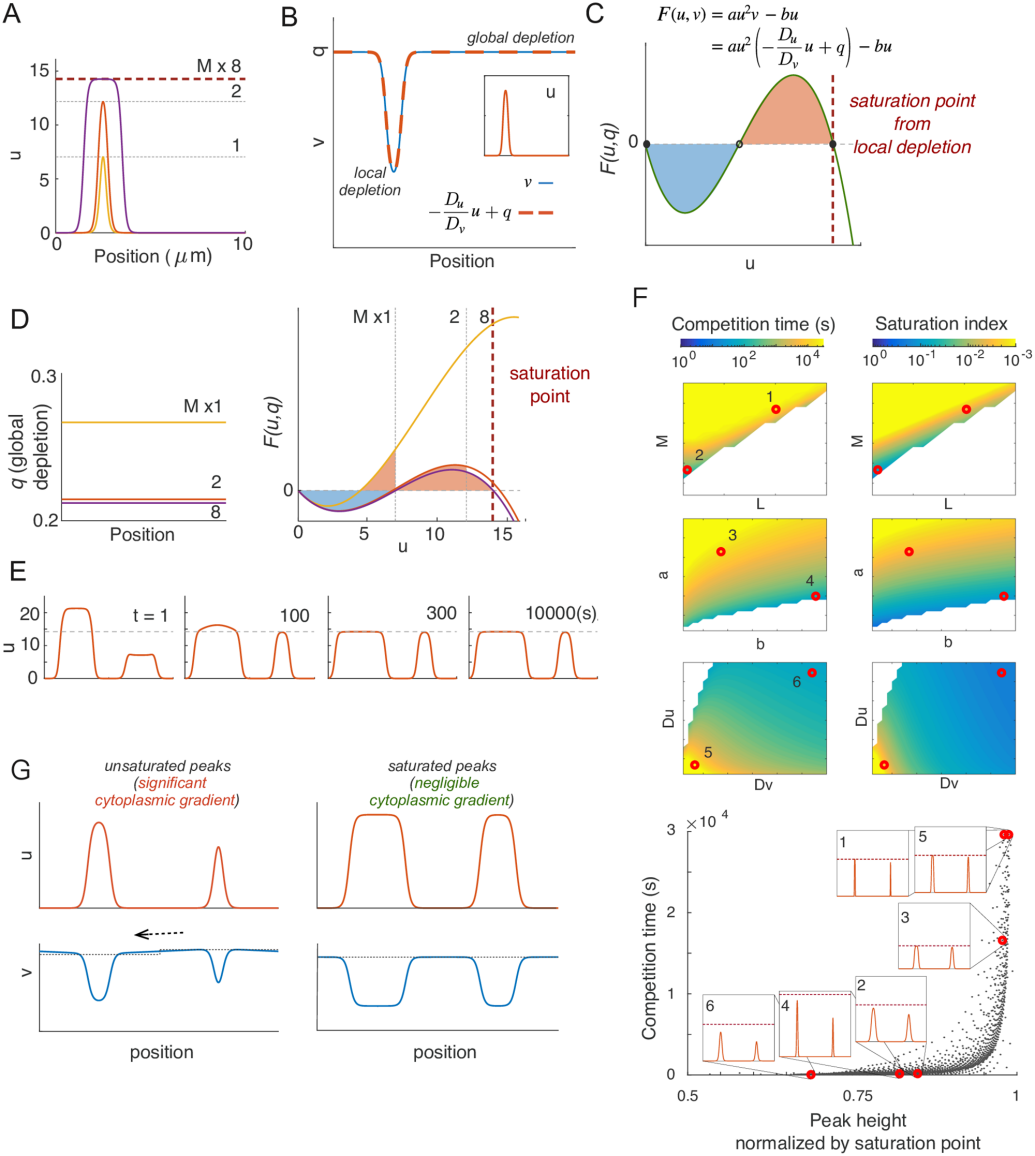
Local substrate depletion leads to saturation and slow competition. A) Turing-type model with *D*_*v*_ < ∞ displays a transition between unsaturated peaks and saturated mesas with increased *M*. B) Local depletion of *v* in the cytoplasm beneath the peak results in a linear relationship between the concentration profile of *v* and *u*. Inset indicates *u* profile. C) The effect of local depletion transforms the reaction term of the Turing-type model from a quadratic *F*(*u*, *v*) to a cubic *F*(*u*, *q*), yielding a third fixed point. D) The cubic reaction term *F*(*u*, *q*) results in a behavior similar to that of the wave-pinning model: When *M* is low, *q* is high, and the peak is sharp; when *M* increases, depletion of cytoplasmic substrate makes *F*(*u*, *q*) drop, and *u*_max_ eventually approaches a saturation point. E) Peaks saturated by local depletion are meta-stable. F) Saturation index correlates with competition timescale. Simulations and display as in Fig 4A and 4B. Parameter variations in *a* vs *b* and *D*_*u*_ vs *D*_*v*_ consist of 30 × 30 simulations each of 0.1× to 3× of the basal parameter values. Parameter variations in *M* vs *L* consists of 15x15 simulations of 0.2× to 3× basal parameter values. Basal parameters are as in Fig 4A, except that *D*_*v*_ = 1*μm*^2^*s*^−1^. Graph shows all simulations plotted as in Fig 4B, with illustrative simulations corresponding to numbered red dots. G) When *D*_*v*_ is finite, the basal cytoplasmic substrate concentration underneath each peak (shown in dashed lines) quickly reaches a quasi-steady state with the recruitment power of the peak. The stronger the recruitment power of the peak, the lower the basal cytoplasmic substrate level. This creates a cytoplasmic gradient when two peaks have different recruitment power, resulting in a cytoplasmic flux towards the larger peak. The gradient becomes negligible when both peaks are saturated, resulting in meta-stable peaks.

When cytoplasmic diffusion is finite (*D*_*v*_ < ∞), a saturation point emerges even if there is no explicit saturation in the reaction term. With finite *D*_*v*_, increasing *M* in the Turing-type model (Eq 4) yields saturated mesas that become broader as *M* increases (Fig 5A), similar to that seen with the wave-pinning model (Eq 5).

To understand this behavior, recall that at steady state, (Eq 3) must hold. Adding (Eq 3a) and (Eq 3b), integrating and enforcing the periodic boundary condition yields a linear relationship between *u* and *v*, regardless of the reaction term:

$$\begin{array}{c} v_{ss}=-\frac{D_{u}}{D_{v}}u_{ss}+q \end{array}$$ (7)

where *q* is a constant over space that is depleted globally over time analogous to *v* in the *D*_*v*_ → ∞ limit. This reflects the fact that in addition to global substrate depletion, activation due to positive feedback depletes *v* locally under a peak of *u*, creating a “dip” in the concentration of the cytoplasmic GTPase *v* that corresponds to the peak of *u* in a linear manner (Fig 5B).

Local depletion results in an emergent saturation effect, because substituting Eq 7 into the reaction term of the Turing type model (Eq 4) gives:

$$\begin{array}{c} F\left(u_{ss},q\right)=au_{ss}^{2}(-\frac{D_{u}}{D_{v}}u_{ss}+q)-bu_{ss} \end{array}$$ (8)

This new reaction term *F*(*u*, *q*) is a cubic in *u*, and can have three fixed points (Fig 5C). The upper fixed point reflects the *u* concentration at which local depletion of *v* precisely balances the net recruitment of *u*, yielding an emergent saturation point. Thus, even when there is no saturation inherent in the reaction term of the model, local depletion of *v* under the peak acts to limit local production of *u*, introducing a saturation effect. Given sufficient total mass *M*, *u*_max_ approaches this saturation point, resulting in a saturated mesa for reasons described above with the wave-pinning model (Fig 5D). In this case, it is possible to derive a simple expression for the saturation point (For details, see Supporting Information section 2):

$$\begin{array}{c} u_{\text{sat}}=\sqrt{\frac{2bD_{v}}{aD_{u}}} \end{array}$$ (9)

As with saturation due to the wave-pinning reaction term, saturation by local depletion also slowed competition dramatically, leading to meta-stable peaks (Fig 5E). Exploration of a wide parameter range indicated that as with saturation via the reaction term, saturation due to local depletion of substrate is also a dominant contributor to the timescale of competition (Fig 5F).

When *D*_*v*_ < ∞, two unequal peaks no longer “see” the same level of substrate, *v*. Instead, the local *v* rapidly reaches a quasi steady-state with each peak (Fig 5G). When two unsaturated peaks coexist, the higher peak has a stronger recruitment power for reasons discussed in Fig 2F. This drives a greater depletion and hence lower baseline of *v* under the higher peak, generating a cytoplasmic *v* gradient that drives a flow of GTPase towards the higher peak, and hence competition (Fig 5G). In contrast, when two unequal but saturated peaks coexist, they have similar recruitment power, so there is a negligible cytoplasmic gradient, and competition occurs on a dramatically slower timescale.

### Effect of increasing distance between peaks

**Fig 6:**
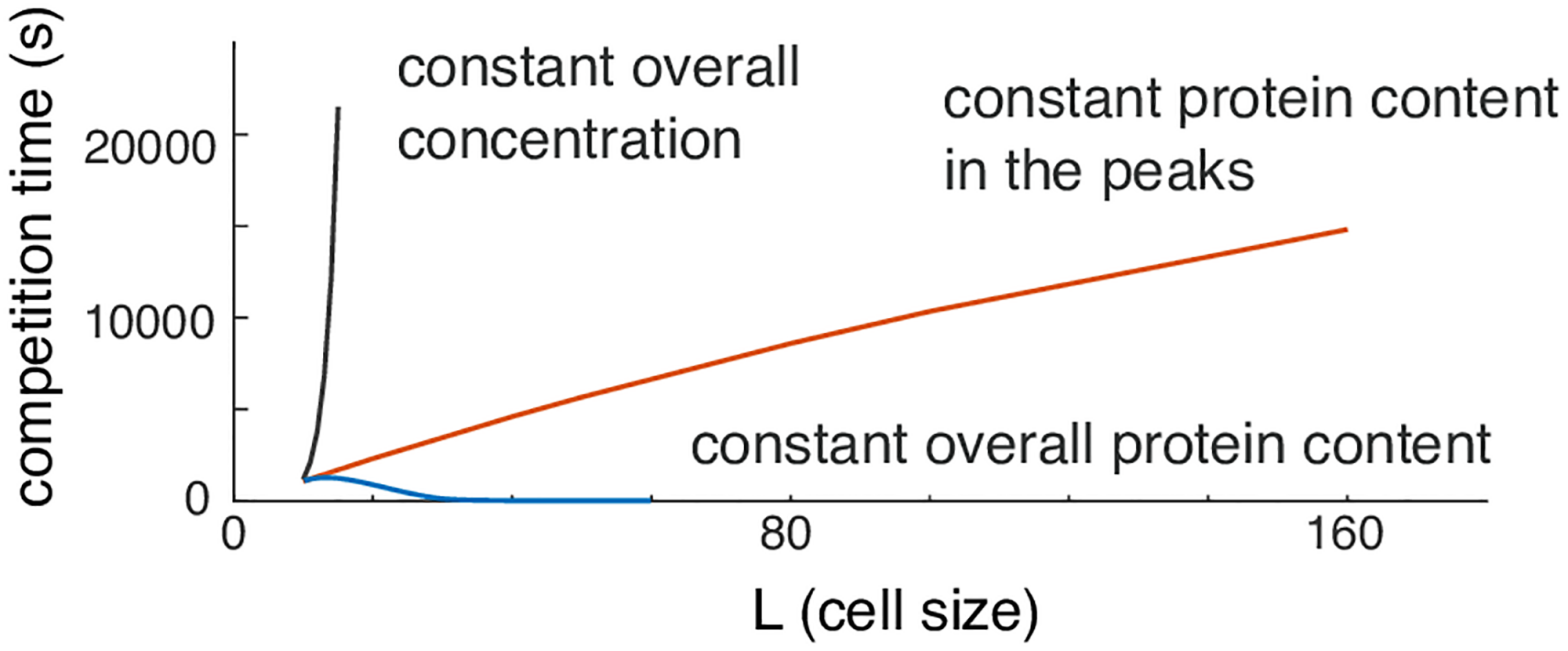
Effect of domain size on competition time. Effect of expanding the domain size on competition time. Gray: overall concentration was held constant as L increases (proportional increase of total protein content in the system *M*; peaks saturate). Blue: overall protein content constant (peaks shrink to feed the larger cytoplasm). Red: protein content in the peaks is maintained constant (identical peak shape).

During competition, GTPase is transferred from the “losing” peak to the “winning” peak through the cytoplasm. Thus, increased distance between the peaks or a decreased diffusion constant in the cytoplasm would be expected to slow the transfer and hence slow competition (an effect not seen when *D*_*v*_ → ∞). To assess how effective increased distance could be in slowing competition, we initially considered the effect of increasing cell size while keeping overall GTPase concentration constant (Fig 6, gray line). Competition slowed dramatically as domain size *L* was increased, but this does not distinguish whether increasing distance between peaks or increasing total GTPase content *M* (moving the peaks closer to saturation) is responsible for the slowing of competition. Increasing *L* without changing *M* resulted in GTPase dilution and hence smaller peaks that competed more rapidly despite the increased distance between peaks (Fig 6, blue line). To maintain equivalent peaks, we increased *L* while adding the exact amount of GTPase required to fill the cytoplasm in the extended domain so that the amount of GTPase *in the peaks* remained constant. This scenario allowed us to quantify the effect of increasing distance between peaks without confounding changes in peak size. The result was that competition became slower in a sub-linear manner with distance (Fig 6, red line). Thus, distance between peaks can slow competition, but does so in a much more gradual manner than the approach to saturation.

### Other MCAS models also link competition timescale to saturation

**Fig 7:**
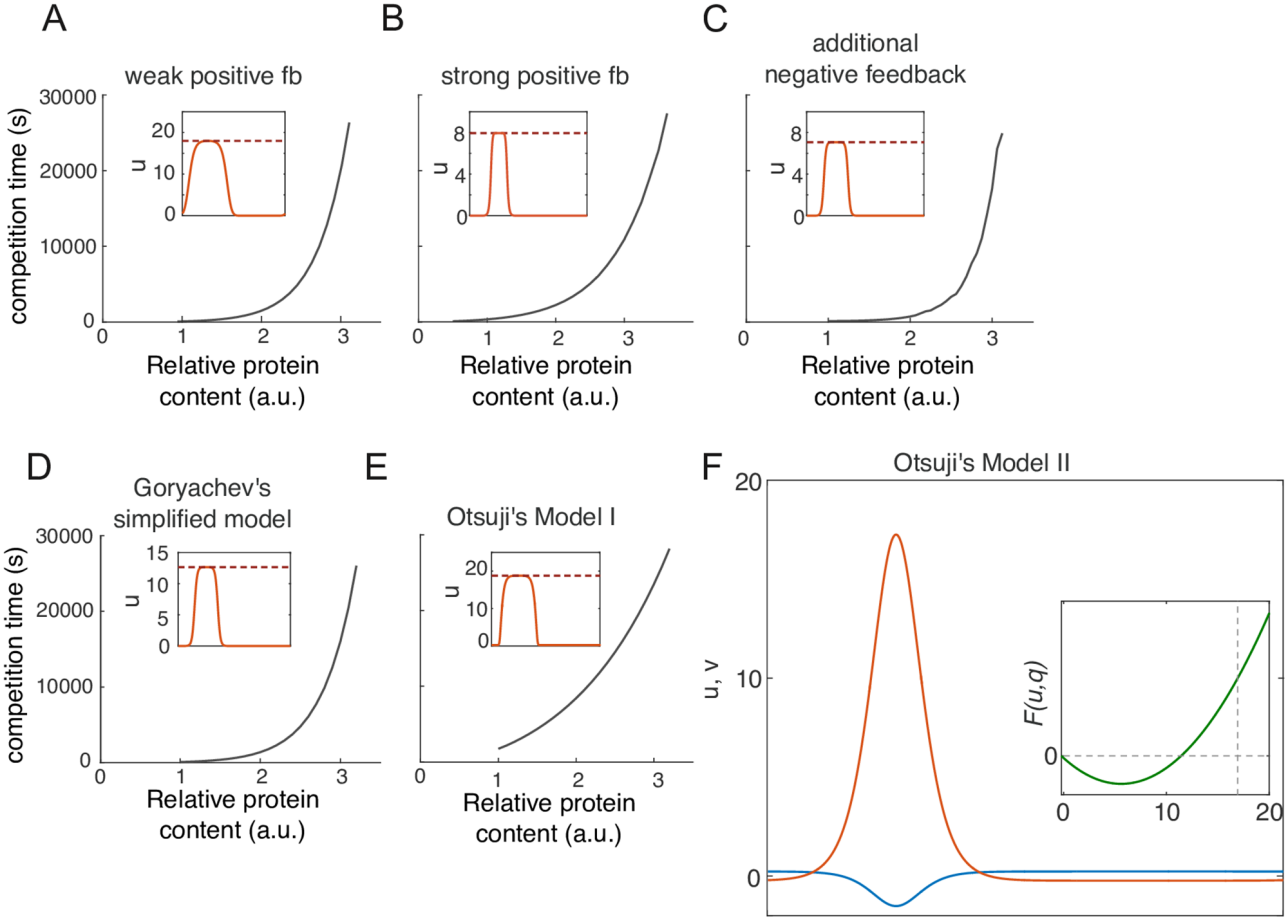
Other MCAS models also link competition timescale to saturation. Competition time increase dramatically with increased total protein content (*M*) in other MCAS models. Insets: peak shape upon reaching saturation. Red dashed lines indicate saturation point. A) weak positive feedback, *F*(*u*, *v*) = *u*^1.2^*v* − *u*; B) strong positive feedback, *F*(*u*, *v*) = *u*^3^*v* − *u*; C) additional negative feedback *F*(*u*, *v*) = (*u*^2^ − 0.01*u*^4^)*v* − *u*; D) Goryachev’s simplified model *F*(*u*, *v*) = (*u*^2^ + *u*)*v* − *u* [9]; E) Otsuji’s model 1 *F*(*u*, *v*) = *a*_1_*v* − *a*_1_(*u* + *v*)/[*a*_2_(*u* + *v*) + 1]^2^ with the original parameters described in [11]. In each instance, competition time slows down dramatically as peaks saturate. F) In Otsuji’s model 2 with the original parameters, *F*(*u*, *v*) = *a*_1_(*u* + *v*)[(*D*_*u*_/*D*_*v*_*u* + *v*)(*u* + *v*) − *a*_2_] [11], saturation is avoided by allowing negative values of *u* or *v*.

Our analysis has focused on specific illustrative models, but many other forms of *F*(*u*, *v*) in Eq 2 can also support polarization. For example, positive feedback strength may vary, yielding different exponents for the activation term (e.g. *f*(*u*) = *u*^1.2^ with weak feedback, or *f*(*u*) = *u*^3^ with strong feedback). Or, positive feedback may operate by reducing inactivation rather than by increasing activation (e.g. *f*(*u*) = 1, *g*(*u*) = *u*/(1 + *u*^2^)). Or, positive feedback may be accompanied by negative feedback, as proposed for the yeast polarity circuit [17, 23] (e.g. *f*(*u*) = *u*^2^ − *cu*^3^). As local cytoplasmic depletion is a universal mechanism of saturation, we would expect that competition time slows down as the system approaches saturation in all of these models. Indeed, all of these variations displayed a saturation point, leading to a transition from unsaturated to saturated peaks as *M* was increased. And in each case, the change in peak shape was accompanied by a dramatic slowing of competition (Fig 7A–7E). This suggests that our findings are broadly applicable to MCAS models.

The only counterexample we have encountered so far is model II from [11], where

$$\begin{array}{c} F\left(u,v\right)=a_{1}\left(u+v\right)[(\frac{D_{u}}{D_{v}}u+v)\left(u+v\right)-a_{2}] \end{array}$$ (10)

Unlike other reaction terms based on mass action kinetics (Eq 2), this reaction term is not dependent on *v*, but rather on the combined concentration of *u* and *v*. Thus, activation in this model is no longer restricted by *v* depletion as in the other models mentioned above, and *v* can assume negative values when *u* is high, avoiding saturation (Fig 7F). This eliminates the effect of local depletion: When *v* is substituted with $-\frac{D_{u}}{D_{v}}u+q$, *F*(*u*, *q*) is a curve lacking a third fixed point (and hence lacking saturation). However, as concentrations of *u* or *v* cannot be negative in cells, this model is not physiologically relevant.

### Competition on a 2-dimensional membrane

**Fig 8:**
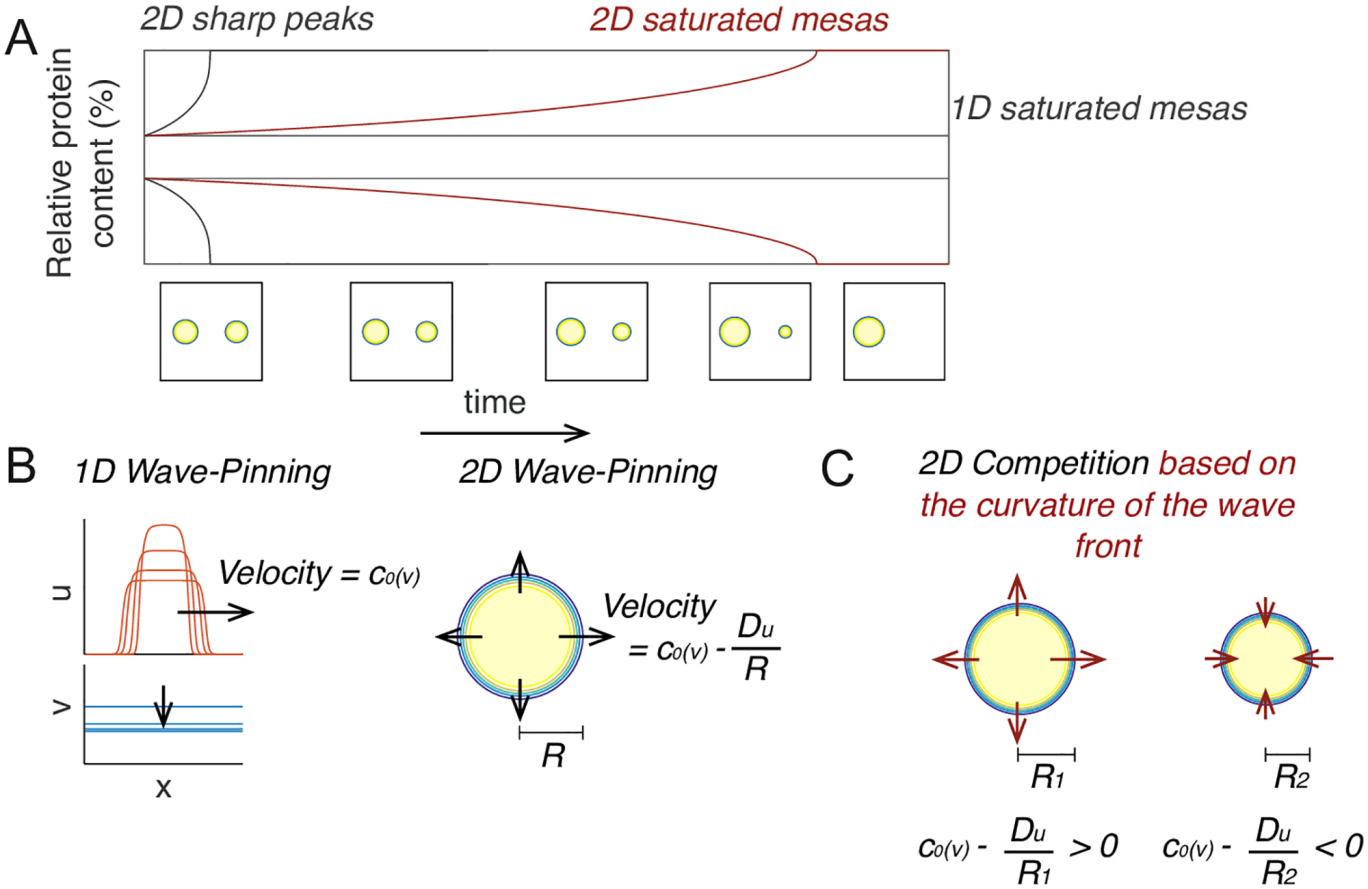
Competition in 2D can be driven by differences in peak curvature. A) In 2D, saturated mesas compete faster than they would in 1D, but slower than unsaturated peaks. B) Left: In the 1D wave-pinning model assuming infinite *D*_*v*_, the velocity of the traveling wave front is a function of *v*. Right: In 2D, the velocity is also dependent on the radius of the mesa, because GTPase diffusing across the wavefront becomes diluted in a manner dependent on the curvature of the wavefront. C) When two unequal mesas coexist in 2D, they share the same cytoplasmic *v* and therefore have the same *c*_0_, but because of the the difference in radii, the larger mesa will expand at the expense of the smaller, leading to competition.

To simplify the analysis, the discussion above focused on competition between peaks in 1D. The conclusions that differences in recruitment power drive competition and that a peak’s recruitment power saturates as peaks become larger both hold in 2D as well as 1D (Supporting Information section 6). However, simulations show that saturated mesas compete on faster timescales in 2D than in 1D (Fig 8A). As discussed below, this is due to a second driving force for competition that depends on the 2D curvature of the peaks.

In wave-pinning models, the edges of growing 1D mesas resemble traveling wave-fronts ([10]). The speed of the traveling wave, *c*_0_, depends on the abundance of cytoplasmic substrate, *v* (Fig 8B). As more *v* is converted to *u*, cytoplasmic *v* is depleted until *c*_0_ drops to zero, at which point the wave is pinned, forming a steady state peak. However, in 2D, a circular wave spreading as a peak grows will have a speed less than *c*_0_, because diffusive spreading of *u* from the front to activate neighboring membrane is diluted by the geometry of the wave front (Fig 8B). Previously developed theory ([24–26]) indicates that in this context the wave speed *c* is dependent on the curvature of the wavefront, *κ*:

$$\begin{array}{c} c=c_{0}\left(v\right)-\kappa D_{u} \end{array}$$ (11)

For a circular peak of radius *R*, $\kappa =\frac{1}{R}$. Thus, smaller peaks with high curvature spread more slowly than otherwise similar larger peaks.

For a situation in which unequal circular mesas coexist in 2D, these considerations show that even if *c*_0_ and *v* are the same for both peaks, the difference in peak curvature suffices to give the larger peak an advantage over the smaller one (Supporting Information section 7). Each peak grows or shrinks depending on whether *c*_0_ is larger or smaller than $\frac{D_{u}}{R}$. As peaks initially develop, *v* is high enough that both peaks can grow, but as *v* becomes depleted, *c*_0_ decreases until the smaller peak transitions to shrinking (Fig 8C). This liberates more *v* so that the larger peak can continue to grow until it is the only peak present. Thus, in 2D there are two drivers of competition between unequal peaks: a difference in recruitment power between peaks of different height, and a difference in curvature between peaks of different radii. Unlike in 1D, the latter can drive competition even for saturated peaks with negligible difference in peak height.

**Fig 9:**
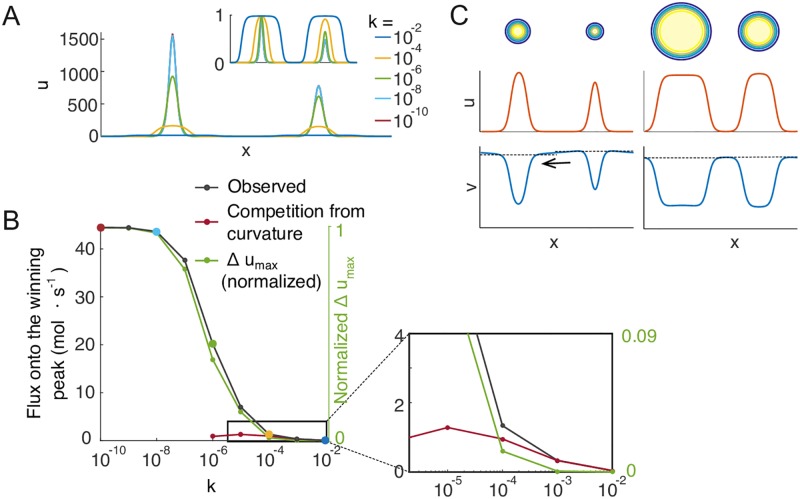
Comparison of competition fluxes driven by differences in peak height or peak curvature in 2D. A) Peaks with 60% and 40% protein content were obtained from simulations of the wave-pinning model (Eq 5) using different values of the parameter *k*. Inset: normalized to *u*_max_. Parameter values: *L* = 10 *μ*m; *a* = *b* = 1 *s*^−1^; *D*_*u*_ = 0.01 *μm*^2^*s*^−1^; *k* as labeled by color. B) Simulating competition between the peaks illustrated in A), the net protein transfer (gray line) tracks closely with the differences in peak height when peaks are not saturated (green line, normalized to the flux at *k* = 10^−10^). As peaks approach saturation (region expanded in the inset), competition fluxes no longer track with the difference in peak height, and instead approach the fluxes predicted by curvature-driven competition (red line, see Supporting Information section 7). Fluxes driven by differences in peak height are much larger than those driven by differences in peak curvature. C) The Turing-type model Eq 4 was simulated in 2D with finite cytoplasmic diffusion yielding emergent saturation. As in 1D, unsaturated peaks generate a steeper cytoplasmic GTPase gradient than saturated mesas, yielding much faster competition.

Although saturated mesas are able to compete in 2D, simulation results suggest that such competition is slow relative to that between unsaturated peaks (Fig 8A). To systematically compare competition rates for different peaks, we calculated the net flux of GTPase from the losing peak to the winning peak, expressed as mole GTPase per second. As the flux changes over the course of competition, we chose the point at which the winning peak had 60% and the losing peak had 40% of the total GTPase in the peaks. We first consider the limit where *D*_*v*_ is infinite, and use the wave-pinning model to generate saturated or unsaturated peaks. We kept all parameters including total GTPase *M* constant and varied the parameter *k* (Eq 5) to generate peaks of different shapes but similar GTPase content (Fig 9A). This revealed that competition fluxes were much larger for peaks that were far from saturation than for saturated peaks (Fig 9B). For saturated peaks, the fluxes matched those predicted for curvature-driven competition (see Supporting Information section 7) (Fig 9B, red line). However, when peaks were no longer in the saturated regime, the fluxes diverged from the prediction for curvature-driven competition, and were approximately proportional to the differences in peak height (Fig 9B, green line). These results indicate that when peaks are saturated, competition is driven by curvature, whereas when peaks are not saturated, competition fluxes become significantly larger and competition is primarily driven by difference in peak height, as in 1D.

Similar results were obtained simulating Turing-type models with finite cytoplasmic diffusion, where saturation emerges as a consequence of local *v* depletion. As in 1D, competing peaks far from saturation generated a significant cytoplasmic gradient of *v* driving large fluxes of GTPase, while saturated mesas did not (Fig 9C). These observations suggest that although peak curvature contributes to competition in 2D, it provides a relatively weak driving force. The dominant factor for competition timescale is still the difference in recruitment power, which decreases rapidly as peaks approach saturation.

## Discussion

### A “saturation rule” underlies the difference between uni- and multi-polarity

Since Turing’s landmark 1952 paper [13], the power of two-component reaction-diffusion models to generate a variety of spatial patterns has fascinated mathematical biologists. Early models with slowly-diffusing activators and rapidly-diffusing substrates formed activator peaks with a spacing dictated by a characteristic wavelength [12]. However, addition of a constraint specifying that the total mass of activator and substrate in the system be conserved led to the finding that some such MCAS systems evolved over time from multipolar to unipolar outcomes with a single peak of activator [9, 11, 17, 18]. Our results suggest that all MCAS models would, given sufficient time, yield unipolar outcomes.

Earlier studies on MCAS systems emphasized that different models can display different behaviors, including Turing instability and wave-pinning dynamics. However, as illustrated here, there is not a categorical distinction between model types. Our results, like recent reports [15, 27, 28], show that a bistable model that yields classical wave-pinning behavior in one parameter regime can also exhibit Turing instability with different parameters. We further show that even a model without explicit bistability conferred by the reaction term will exhibit saturation in a similar way as bistable systems saturate to a fixed point due to local cytoplasmic depletion (see Supporting Information section 5).

In the parameter regimes examined by previous studies, Turing-type model peaks displayed rapid competition, while wave-pinning model peaks coexisted, suggesting that competition might be linked to model architecture ([22, 29]). The propensity of a model to develop uni- or multi-polar profiles has been analyzed by assessing the stability of the homogeneous steady state ([16]), but because competition between peaks occurs far from the homogeneous steady state, those analyses could not predict the outcome of competition. Here, we examined the linear stability of the *two-peak steady state*, and found that all two-peak states in MCAS models are unstable to competition. That is, the larger peak will always grow at the expense of the smaller (scenario 1 in Fig 2A), for all biologically relevant MCAS models.

Although competition will always yield a single-peak outcome given sufficient time, the timescale of competition can vary enormously. Our findings lead us to propose that whether competition yields uni-polar or multi-polar outcomes in a biologically relevant time frame depends primarily on a remarkably simple “saturation rule”. We show that each MCAS model encodes a parameter-dependent saturation point, such that the peak activator concentration saturates at a polarized steady state. As the peak activator concentration approaches the saturation point, the difference in peak height between unequal peaks decreases dramatically, leading to much slower competition and effective coexistence between peaks (scenario 3 in Fig 2A). Varying parameters affects the timing of competition predominantly by affecting the degree to which competing peaks approach the saturation point. Other factors like domain size and wavefront curvature in 2D also influence the dynamics of competition (see below), though to a much lesser degree. Thus, the major novel conclusions from our analysis are that all MCAS models share an ability to saturate, and that the outcome of competition between GTPase clusters depends primarily on whether the model parameters allow the peak GTPase concentrations to approach saturation.

### Competition driven by geometry

In models restricted to one spatial dimension, competition slows exponentially as peaks approach saturation, effectively yielding meta-stable multipolar states. However, on a 2D membrane, a fundamentally different driver of competition emerges in this regime, dependent on the curvature of the wavefront separating regions of high versus low activator concentration. Although much slower than competition between peaks of different activator concentration, this curvature-driven competition allows broader peaks to expand at the expense of narrower peaks. This behavior is similar to that observed in a separate but related class of phase separation models explored by Gamba and colleagues [30–32]. In these models, an activator (for example PIP_3_) can be generated from a substrate (PIP_2_) and vice versa by the action of regulatory enzymes. Due to positive feedback, a membrane develops patches with high PIP_3_ and low PIP_2_ concentration in a membrane with high PIP_2_ and low PIP_3_ concentration. Unlike with the MCAS systems discussed here, the *local* sum of PIP_3_ and PIP_2_ concentration is assumed to be constant. Such systems display a coarsening behavior analogous to the competition between peaks in MCAS systems, and a physical analogy has been drawn to the process of precipitation from supersaturated solution [32]. As with curvature-driven competition in MCAS models, this coarsening is driven by a geometry-dependent “surface tension” feature that makes smaller patches of PIP_3_ prone to dissolve while larger patches grow. Comparison of the kinetics of competition in a phase separation model compared to a MCAS model suggested that, as for the curvature-driven competition we analyzed, competition in the phase separation system was very slow relative to that in an unsaturated MCAS system [29].

### Biological implications of the saturation rule

The models considered in this report represent a drastically simplified system compared to any biological system. Two simplifying assumptions are particularly noteworthy. First, because polarization phenomena often employ stable proteins and occur on rapid timescales compared to cell growth, MCAS models assume a constant domain size and a constant protein amount. This may not always apply. Second, we modeled two-component systems, whereas all known polarity systems have multiple components. More realistic multi-component models of the budding yeast polarity circuit exist [7, 9, 18, 23] and preliminary simulations indicate that they too behave according to the saturation rule. However, adding additional components can yield emergent behaviors not seen in the two-component systems [33, 34]. Thus, predictions of the saturation rule will need to be tested experimentally to assess whether the insights derived from simple MCAS models are translatable to biological systems.

The saturation rule suggests several possible pathways by which a single Rho-GTPase module can regulate the number of polarity sites. A cell may directly regulate the saturation point by tweaking the major mechanism of saturation in each system. If saturation is predominantly due to local depletion of cytoplasmic substrate, then increasing the strength of positive feedback (e.g., increased GEF activity) would lead to more severe depletion, a lower saturation point, and hence slower competition and a multi-polar outcome. Alternatively, if saturation is predominantly due to an additional negative feedback (Fig 7C), then strengthening the negative feedback would lead to a lower saturation point, slowing competition and favoring a multi-polar outcome. The saturation rule thus generates hypotheses specific to each system that can be tested experimentally.

The most obvious prediction perhaps is that systems should transition between uni- and multi-polarity regimes as total GTPase contents change: lower levels should yield uni-polarity, while higher levels sufficient to allow activator concentrations to approach the saturation point should yield multi-polarity. In the tractable budding yeast *Saccharomyces cerevisiae*, the master polarity regulatory GTPase, Cdc42, becomes concentrated at polarity sites, and initial peaks compete on a 1 minute timescale to leave only one winning peak. However, attempts to assess whether raising polarity factor concentrations would yield more peaks were complicated by the fact that overexpression can block polarization [35], presumably because active GTPase spreads throughout the cell cortex. This phenomenon has been explored in Turing models: when component concentrations are too high, the system no longer polarizes, but instead evolves to a stable steady state with high levels of activator uniformly distributed all over the surface [17].

One way to avoid uniform activation is to increase cell volume as well as total protein content in parallel, maintaining overall concentrations unchanged, which is analogous to the gray line in Fig 6. Yeast cells occur naturally as haploids and diploids, and cells with higher ploidy can be constructed. It is also possible to block cytokinesis, generating larger cells due to failed cell division. It appears that cell volume and total protein amount scale with ploidy for most proteins, so that total protein concentrations remain generally unchanged ([36]). If we were to keep the activator and substrate concentrations at the homogeneous steady state of an MCAS model constant, then a model with a larger domain size would provide a larger pool of substrate, allowing greater local enrichment of the activator, so that peak activator concentrations would approach the saturation point. This predicts that as cells become larger they should eventually switch from uni- to multi-polarity.

For some filamentous fungi, like *Ashbya gossypii*, development proceeds through a cell enlargement process in which a single shared cytoplasm houses more and more nuclei. This provides a natural system that samples a large range of cell sizes. Cell polarity in *A. gossypii* is thought to be governed by the same Cdc42-centered circuit employed in *S. cerevisiae*, but these cells transition from always having a single polarity site when they are small (following germination), to having two (and then more) polarity sites as they grow larger, leading to hyphal branching [37]. Sporadic septation (division separating parts of a single large cell into two smaller ones) can restore a single polarity site to the cell, but continued growth then leads to additional polarity site(s) again. This behavior is consistent with a switch from uni- to multi-polarity according to the saturation rule. A prediction for this system would be that reducing total content of polarity proteins should delay the switch from uni-polar to multi-polar behavior, so that it would take a larger cell to initiate a hyphal branch.

### Conclusion

We have examined the behavior of a family of simplified mathematical models that capture key aspects of the behavior of the Rho-GTPases that regulate the formation of cortical domains in cells. Our analysis suggests that all biologically relevant models of this type (and there are several varieties) display reproducible transitions in system behavior as parameters vary. In particular, each model has a saturation point that depends on model parameters. With low amounts of GTPase, the system forms sharp peaks of active GTPase, but as GTPase levels increase, the peak GTPase concentration approaches the saturation point and the concentration profiles broaden into saturated mesas. If there are two or more peaks of GTPase, the peaks will compete with each other until one emerges as the single stable winner. However, the time scale of competition slows dramatically as the peaks broaden, so in practice the systems transition from a situation with rapid cut-throat competition to one in which competition is so languid that peaks coexist on biologically relevant timescales. Local depletion of the cytoplasmic substrate provides a mechanism of saturation that is universal to all activator-substrate systems, so regardless of the specific biochemical feedback mechanism, a cell that polarizes through local activation and substrate depletion should be able to switch between uni- or multi-polar outcomes by regulating system parameters. The discovery of this intrinsic property of the Rho-GTPase system suggests hypotheses testable in the context of various different cell types.

## Methods

### Model simulation

Simulations of the MCAS models were done on MATLAB with parameters described in the main text. One-dimensional simulations were done on domains with fixed spatial resolution of 500 grid points, except the simulations with large domains, where number of grid points was increased proportionately. Finite differences were used with the linear diffusion being treated implicitly and the nonlinear reaction term explicitly in the time stepping. Two-dimensional simulations are computed on 200 × 200 grid points implemented using implicit spectral methods. For all simulations in the limit *D*_*v*_ → ∞, the mean of *v* was taken every time step. All simulations proceeded with adaptive time stepping according to relative error in the reaction term. The MATLAB code used for simulations is provided in Source Code Files.

### Calculation of competition time

Simulations of competition is generally generated as follows: Two-peak steady states were first generated by simulating the evolution of the homogeneous steady state with an added sine wave. Perturbations were then introduced by increasing the amplitude of the concentration profiles *u*(*x*) *v*(*x*) at the first peak by a given percentage (e.g. a 20% increase), and decreasing the amplitude of the second peak by the same percentage (e.g. a 20% decrease). The resulting two unequal peaks were then allowed to compete.

For simulations used in Figs 4A and 5F, we recorded the measurements of the peak height (*u*_max_) to calculate the saturation index, and the competition time. The steady state *u*_max_ was obtained from the two-peak steady state. The two peaks were then perturbed by increasing the protein content of the left half-domain and decreasing the protein content of the right half-domain, so that each half has 60% and 40% of the original *M*, respectively. For more accurate measurements of the competition time, the two halves were first simulated individually to their own steady states in isolation. Upon the start of competition, the two half-domain were allowed to communicate through diffusion, and the competition time was calculated by measuring the resolution time of two unequal peaks from 60% and 40% at the beginning to 99% and 1%.

## Supporting information

S1 Text Supporting information. (PDF)

S1 File MATLAB code. (ZIP)
