# Supplementary material for: Principles that govern competition or co-existence in Rho-GTPase driven polarization
Source: PLoS Comput Biol. 2018 Apr 12;14(4):e1006095. doi: 10.1371/journal.pcbi.1006095 (PMC5916526; doi:10.1371/journal.pcbi.1006095)
Supplement: S1 Text — (PDF) [file pcbi.1006095.s001.pdf]

Supporting Information

1 Non-dimensionalization of 1D MCAS Models

We consider the mass-conserved activator-substrate (MCAS) model in a one-dimensional domain with periodic boundary conditions,

$$\frac{\partial u}{\partial t} = D_u \frac{\partial^2 u}{\partial x^2} + F(u, v) \tag{1a}$$

$$\frac{\partial v}{\partial t} = D_v \frac{\partial^2 v}{\partial x^2} - F(u, v) \tag{1b}$$

where the diffusion of v is much faster than u, as set by $D_v \gg D_u$.

This model is a mass-conserved version of an activator-substrate model, where u is the activator and v is the substrate. As the activator concentration u increases at certain locations, the substrate v is depleted at the same rate, therefore, the mass M is a constant conserved for all time,

$$M = \int_0^L (u + v)\, dx. \tag{2}$$

The reaction terms of these models, given by $F(u, v)$, generally contain a v-dependent activation term with nonlinear positive-feedback, and a v-independent inactivation term.

$$F(u, v) = f(u)v - g(u) \tag{3}$$

For example, the model proposed by [1] has an $f(u)$ with a saturable non-linear term and a linear $g(u)$,

$$F(u, v) = \left(k_0 + \frac{au^2}{K^2 + u^2}\right) v - bu \tag{4}$$

We assume negligible k_0, and rewrite this system with dimensionless variables $\tilde{u}$, $\tilde{v}$, $\tilde{x}$, $\tilde{t}$, by scaling the length by the domain size L, and time by T and u and v by U,

$$\tilde{x} = \frac{x}{L}, \qquad \tilde{t} = \frac{t}{T}, \qquad \tilde{u} = \frac{u}{U}, \qquad \tilde{v} = \frac{v}{U}$$

yielding

$$\frac{\partial \tilde{u}}{\partial \tilde{t}} = \frac{D_u T}{L^2} \frac{\partial^2 \tilde{u}}{\partial x^2} + \tilde{F}(\tilde{u}, \tilde{v}) \tag{5a}$$

$$\frac{\partial \tilde{v}}{\partial \tilde{t}} = \frac{D_v T}{L^2} \frac{\partial^2 \tilde{v}}{\partial x^2} - \tilde{F}(\tilde{u}, \tilde{v}) \tag{5b}$$

where the nondimensionalized reaction terms are now

$$\tilde{F}(\tilde{u},\tilde{v}) = \frac{T}{U}\left(a\frac{U^2\tilde{u}^2}{K^2+U^2\tilde{u}^2}U\tilde{v} - bU\tilde{u}\right)$$

and the non-dimensional mass $\tilde{M}$ is

$$\tilde{M} = \frac{M}{U} = \int_0^1 (\tilde{u}+\tilde{v})\,d\tilde{x}$$

Setting the timescale and concentration scale as

$$T = \frac{1}{b}, \qquad U = K\sqrt{\frac{b}{a}}$$

and dropping the tildes, puts the system into the form

$$\frac{\partial u}{\partial t} = \delta\frac{\partial^2 u}{\partial x^2} + F(u,v) \tag{6a}$$

$$\frac{\partial v}{\partial t} = \eta\delta\frac{\partial^2 v}{\partial x^2} - F(u,v) \tag{6b}$$

where

$$F(u,v) = \frac{u^2}{1+\kappa u^2}v - u \tag{7}$$

and the dimensionless parameters are

$$\delta = \frac{D_u}{bL^2}, \qquad \eta = \frac{D_v}{D_u}, \qquad \kappa = \frac{U}{K} = \frac{b}{a}.$$

Setting κ to zero, we obtain a "Turing-type" system with

$$F(u,v) = u^2 v - u. \tag{8}$$

2 Steady state solutions of 1D MCAS models for the $D_v \gg D_u$ limit

We first consider the simplified case where $\eta \to \infty$. The solutions u, v can be expanded as regular perturbation series with respect to inverse powers of η,

$$v = v_0 + \frac{1}{\eta}v_1 + O(\eta^{-2}) \qquad u = u_0 + \frac{1}{\eta}u_1 + O(\eta^{-2}), \tag{9}$$

and substituted into (6). The leading order equation for u_0 at $O(1)$ mirrors (6a),

$$\frac{\partial u_0}{\partial t} = \delta\frac{\partial^2 u_0}{\partial x^2} + F(u_0,v_0), \tag{10a}$$

while at $O(\eta)$, the leading order equation for v_0 becomes $v_{0,xx} = 0$. Subject to the periodic boundary conditions, this forces v_0 to be spatially uniform, but it can depend on time, $v_0 = v_0(t)$. In order to obtain an equation defining the evolution of v_0 we proceed to the next term in the expansion. At $O(1)$ we find that in order for a solution for v_1 to exist, the inhomogeneous terms in the equation must satisfy a solvability

condition. Set by the Fredholm alternative theorem, this condition gives the evolution for $v_0(t)$ in terms of the reaction rate averaged over the domain,

$$\frac{dv_0}{dt} = -\int_0^1 F(u_0, v_0)\, dx. \tag{10b}$$

This equation effectively describes the evolution of the average substrate concentration in the well-mixed limit. Moving forward, we will drop the zero-subscripts and focus on solving this leading order system.

At steady state, the solution $(u_{ss}(x), v_{ss})$ satisfies the system of equations

$$0 = \delta \frac{d^2 u_{ss}}{dx^2} + F(u_{ss}, v_{ss}) \tag{11a}$$

$$0 = -\int_0^1 F(u_{ss}, v_{ss})\, dx \tag{11b}$$

and the constraint on the total mass

$$M = v_{ss} + \int_0^1 u_{ss}\, dx. \tag{12}$$

To understand the properties of the solutions it is helpful to integrate (11a) with respect to du to obtain

$$\begin{aligned} H &= \int \delta \frac{d^2 u_{ss}}{dx^2}\, du + \int F(u_{ss}, v_{ss})\, du \\ &= \int \delta \frac{d^2 u_{ss}}{dx^2} \frac{du}{dx}\, dx + \int F(u_{ss}, v_{ss})\, du \\ &= \frac{\delta}{2} \left(\frac{du_{ss}}{dx} \right)^2 + \int F(u_{ss}, v_{ss})\, du \\ &= E_k + \Phi(u_{ss}, v_{ss}) \end{aligned} \tag{13}$$

where H is a constant and

$$E_k = \frac{\delta}{2} \left(\frac{du_{ss}}{dx} \right)^2 \qquad \Phi(u_{ss}, v_{ss}) = \int F(u_{ss}, v_{ss})\, du.$$

Note that (11a) can be interpreted as the equation of motion for a particle moving in 1D with δ playing the role of mass, x the role of time, and $-F(u_{ss}, v_{ss})$ the applied force. H can then be interpreted as the total energy, constant of the motion. Therefore, solutions for u_{ss} represent periodic solutions for a 1D conservative system.

The local extrema, $u_{\min}$ and $u_{\max}$, occur where $du_{ss}/dx = 0$. A direct consequence of this is that for a given value of H, the integral of $F(u, v)$ from $u_{\min}$ to $u_{\max}$ must be zero to satisfy (13) (Fig. 2 BC in the main text). This condition has been referred to as the **wave-pinning condition** [1], and is general to all MCAS models,

$$\Phi(u_{\min}, v) - \Phi(u_{\max}, v) = 0. \tag{14}$$

For bi-stable models, such as the wave-pinning model (7), there exists a value of the cytoplasmic concentration v at which $F(u, v) = 0$ at both $u_{\min}$ and $u_{\max}$,

$$F(u_{\min}, v_{\text{sat}}) = F(u_{\max}, v_{\text{sat}}) = 0. \tag{15}$$

We refer to this condition as the **saturation condition**. When this condition is met, the steady state solution locally approaches uniform concentrations at both $u_{\min}$ and $u_{\max}$ (e.g. Fig. S1, left panel), which is crucial

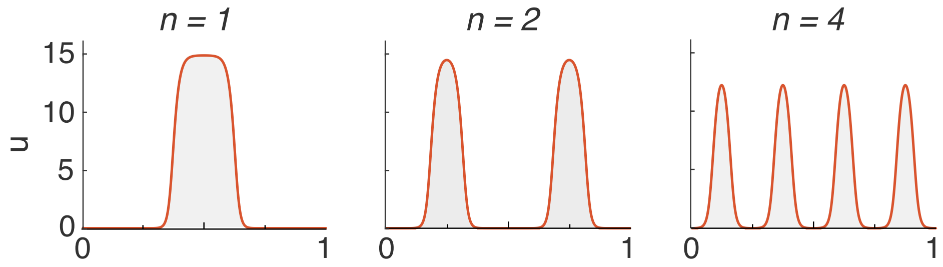

Figure S1: Spatial profiles for steady states with different spatial periods $P = 1/n$**.**
Multipeak solutions with $n = 1, 2, 4$ and the same mass M. Each solution has a different steady state substrate level v.

for later discussion of competition time scale. At saturation, we label the value of $u_{\max}$ as u_{sat}, which is the largest value of $u_{\max}$ possible. The **wave-pinning condition** and the **saturation condition** together are sufficient to solve for v_{sat} and u_{sat} in any given MCAS system.

In a general case where $\eta < \infty$ (finite cytoplasmic diffusion D_v), we can obtain the steady state v profile, $v_{ss}(x)$, as a linear function of $u_{ss}(x)$ by adding (6a and b), integrating twice over x, and enforcing the periodic boundary condition:

$$v_{ss}(x) = -\frac{1}{\eta} u_{ss}(x) + q$$

where q is a constant over x. The steady state system can thus be reduced to one variable in x, analogous to the situation when $\eta \to \infty$, and $u_{ss}(x)$ can be solved similarly. This also allows us to derive the expression of u_{sat} as eq. 9 in the main text.

3 Stability Analysis of Multipeak Steady States

The key question of whether competition happens between two peaks can be answered mathematically by assessing the stability of the two-peak steady state solution $(u_{ss}(x), v_{ss})$. Consider the multi-peak steady state when peak number $n = 2$, a steady state solution $(u_{ss}(x), v_{ss})$ has two identical peaks centered at $x = \frac{1}{4}, x = \frac{3}{4}$. Each peak is reflectionally symmetric about its maximum and the overall solution is also symmetric about $x = \frac{1}{2}$ (see Fig. S2A).

The stability of the two-peak solution is studied by assuming small perturbations of the form

$$u(x,t) = u_{ss}(x) + \epsilon U(x) e^{\lambda t} \qquad v(t) = v_{ss} + \epsilon V e^{\lambda t}$$

where λ and $(U(x), V)$ satisfy the linearized eigenvalue problem

$$\delta \frac{d^2 U}{dx^2} + \partial_u F(u_{ss}(x), v_{ss}) U(x) + V \partial_v F(u_{ss}(x), v_{ss}) = \lambda U(x) \quad (16a)$$

$$-\int_0^1 \partial_u F(u_{ss}(x), v_{ss}) U(x)\, dx - V \int_0^1 \partial_v F(u_{ss}(x), v_{ss})\, dx = \lambda V. \quad (16b)$$

The solution $(u_{ss}(x), v_{ss})$ is unstable if there exists at least one eigenvalue with Re$(\lambda) > 0$.

To approach this question it is sufficient to restrict attention to a particular form of eigenmode, one with $U(x)$ being antisymmetric with respect to $x = \frac{1}{2}$, namely $U(x + 1/2) = -U(x)$. Since $u_{ss}(x)$ is symmetric with respect to $x = \frac{1}{2}$, the integrand of the first integral in (16b) is antisymmetric and hence the integral on the whole domain must vanish. Consequently, for this type of eigenmode, $V = 0$ solves (16b) and the system reduces to

$$\delta \frac{d^2 U}{dx^2} + C(x) U(x) = \lambda U(x) \qquad \text{where} \qquad C(x) = \left. \frac{\partial F}{\partial u} \right|_{u_{ss}(x), v_{ss}}. \quad (17)$$

To solve (17) on the full domain $x \in [0, 1]$ with periodic boundary conditions, it is sufficient to solve the equation on a quarter domain, $x \in [0, \frac{1}{4}]$, with boundary conditions

$$U_1(0) = 0, \qquad U_1'(\tfrac{1}{4}) = 0 \quad (18)$$

or

$$U_2'(0) = 0, \qquad U_2(\tfrac{1}{4}) = 0. \quad (19)$$

The periodic function $U(x)$ is then constructed by extending $U_1(x)$ symmetrically with respect to $x = \frac{1}{4}$, $U_1(\frac{1}{2} - x) = U_1(x)$(Fig. S2B), or by similarly extending $U_2(x)$ anti-symmetrically, $U_2(\frac{1}{2} - x) = -U_2(x)$ (Fig. S2C).

We give a shooting argument to show that there is a positive eigenvalue $\lambda_1 > 0$ for solutions having the form given by (18). Differentiating the steady state equation (11a) with respect to x, we obtain

$$\delta \frac{d^2 u'_{ss}}{dx^2} + \frac{\partial F}{\partial u} u'_{ss} = 0 \tag{20}$$

Therefore, $U(x) = u'_{ss}(x)$ solves (17) with $\lambda = 0$. Note that in this case $u'_{ss}(0) = U(0) = 0$ and $u''_{ss}(\frac{1}{4}) = U'(\frac{1}{4}) < 0$ (there is a finite curvature at $u_{\max}$) and hence the second condition in (18) is not satisfied. If $\lambda > \max(C(x))$, we can re-write (17) as $\delta U'' = (\lambda - C(x)), U > 0$ and $U(x)$ will be monotonely growing and $U'(\frac{1}{4}) > 0$. Since we have constructed solutions achieving positive and negative values for $U'(\frac{1}{4})$, by continuity, there exists an eigenvalue in the range $0 < \lambda_1 < \max(C(x))$ that will yield an eigenmode satisfying (18)(Fig. S3A). Similarly, there exists a second eigenmode of the $U_2(x)$ (19) form with eigenvalue in the range $0 < \lambda_2 < \max(C(x))$ (Fig. S3B).

To summarize, there exist $\lambda_1, \lambda_2 > 0$, thus the two-peak steady state is not stable. Further, the eigenfunction U_1 corresponds to one peak growing and the other shrinking, i.e. competition (Fig S3A). The eigenvalue λ_1 corresponds to the timescale for competition. On the other hand, the eigenfunction U_2 corresponds to neighboring sides of each peak growing while the other sides shrink such that peaks merge with each other. The eigenvalue λ_2 corresponds to the timescale for merging (Fig S3B).

4 The eigenvalues for competition and merging

As two-peak steady states are always unstable, the distinction between competition and coexistence of two peaks (Fig3C-E in the main text) does not reflect a change in stability, but rather, a change in the time scale on which competition occurs. As it has been reported that mesas are meta-stable, we inquired how the eigenvalues of competition and merging change with increasing width of the peaks in a two-peak steady state. We will show that

$$\lambda_{\text{compete}} \approx A_1 e^{-\sqrt{\frac{c}{\delta}}\ell_{\text{mesa}}} \qquad \lambda_{\text{merge}} \approx A_2 e^{-\sqrt{\frac{1}{\delta}}\ell_{\text{valley}}} \tag{21}$$

where δ is the diffusion constant of u, and A_1, A_2, c are constants . ℓ_{mesa} and ℓ_{valley} are defined as in Fig S4 with $\ell_{\text{mesa}} + \ell_{\text{valley}} = \frac{1}{2}$. Our derivation proceeds in the following steps:

1. Approximate the steady state as a step function for $C(x)$ in (17), one segment of which is approximated using $u_{\min}$ for and the other using $u_{\max}$. The two segments are connected with a mid-point boundary condition at $x = \ell$ (Fig S4).
2. Calibrate the mid-point boundary condition using the translational eigenmode $U(x) = u'_{ss}(x), \lambda = 0$.
3. Solve the approximated version of (17) for the competition eigenvalue subject to the competition boundary condition (18) and the mid-point boundary condition calculated in the previous step.
4. Similarly, solve (17) for the merging eigenvalue subject to the merging boundary condition (19).

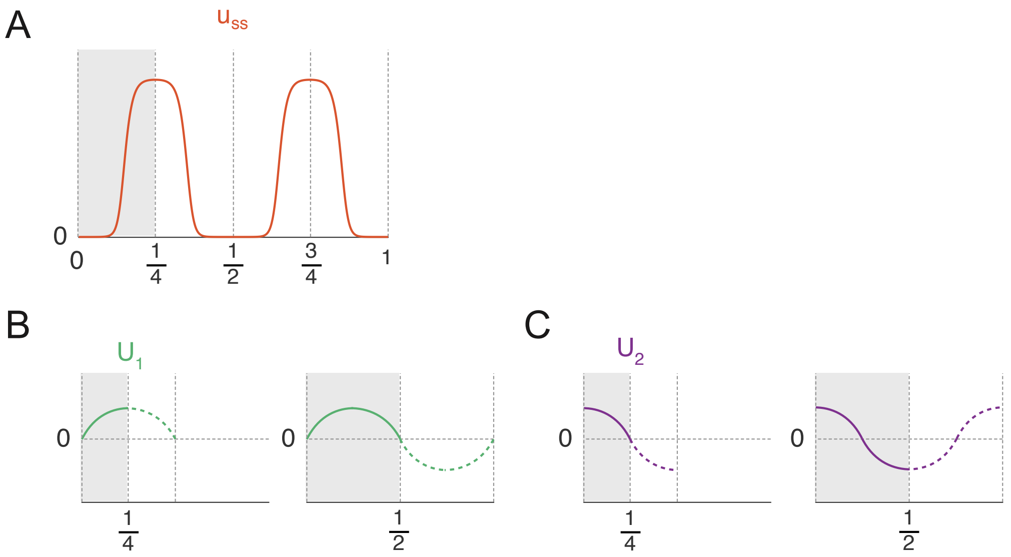

Figure S2: Eigenmodes that solve the eigenvalue problem (16) for a two peak steady state solution. A) A typical two-peak steady state, $u_{ss}(x)$. B, C) Construction of the two forms of the eigenmode $U(x)$ using even and odd extensions of the quarter-domain solutions $U_1(x)$ and $U_2(x)$ for a two-peak solution $u_{ss}(x)$.

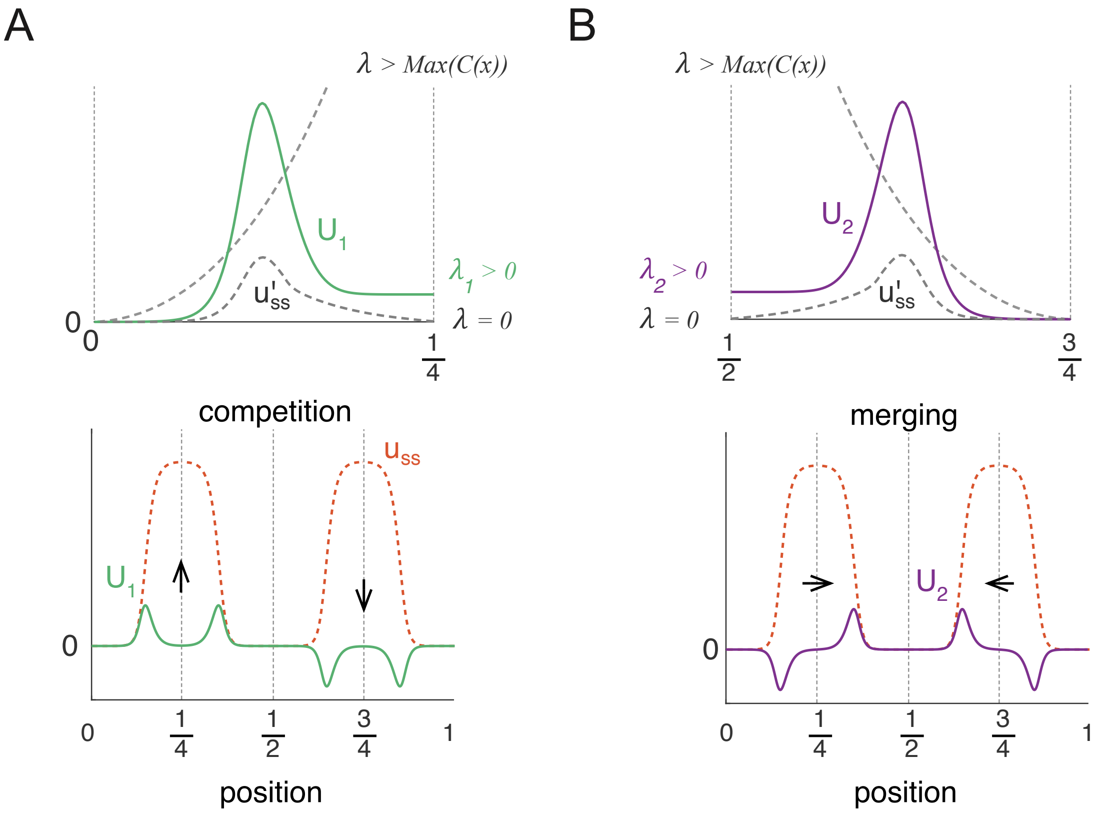

Figure S3: Linear Stability Analysis reveals two unstable modes: competition and merging. The two eigenmodes U_1 and U_2 constructed from the linear stability analysis represents competition (A) and merging (B) of the two peaks in the full domain. The top panel in each gives the two analytic cases (dashed curves) for the shooting argument establishing the existence of the unstable eigenfunction (colored curves). The bottom panel shows the eigenfunction and how it relates to the two-peak steady state profiles.

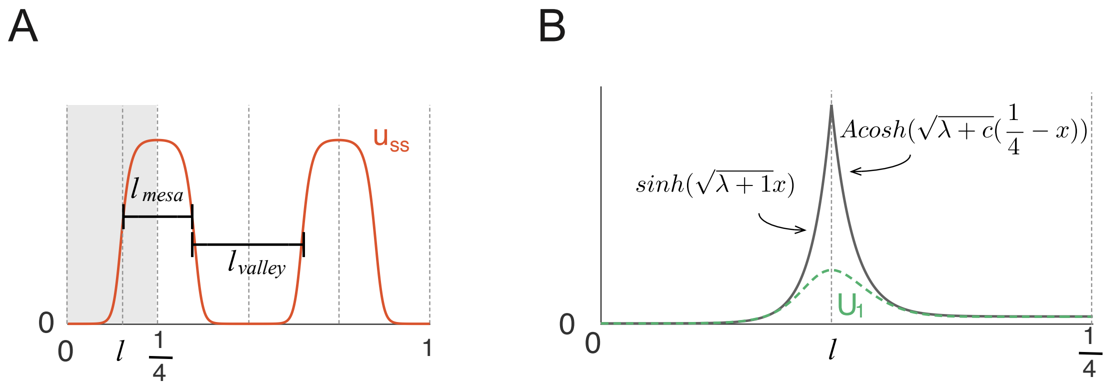

Figure S4: Schematic representation of the near-saturated two-peak steady state.
A) The lengths of the valley and the mesa are equivalent to 2ℓ and $\frac{1}{2} - 2\ell$, with ℓ defined as the position of half max. B) Construction of the approximate $U_1(x)$ eigenfunction using hyperbolic functions with a midpoint boundary condition (24) at position ℓ.

4.1 Approximating the steady state solution

Without loss of generality, let $F(u,v)$ be the non-dimensionalized wave-pinning model (7),

$$F(u,v) = f(u)v - u \qquad \text{where} \qquad f(u) = \frac{u^2}{1+\kappa u^2}.$$

For near-saturation two-peak solutions that have a mesa shape, v is chosen such that (15) is met, thus at $u = u_{\max}$,

$$F(u_{\max}, v) = 0 = f(u_{\max})v - u_{\max} \qquad \Longrightarrow \qquad v = \frac{u_{\max}}{f(u_{\max})}. \tag{22}$$

We try to approximate the eigenvalue problem (17) on a quarter domain by a step function. The two segments $U(x)$ and $\tilde{U}(x)$ satisfy (17) with $u_{ss}(x)$ approximated by $u(x) = u_{\min}$ and $u(x) = u_{\max}$ respectively. The length of U and $\tilde{U}$ are the length of the valley $\ell = \frac{1}{2}\ell_{\text{valley}}$ and the width of the mesa $\tilde{\ell} = \frac{1}{4} - \ell = \frac{1}{2}\ell_{\text{mesa}}$, respectively. As $f'(u_{\min}) = 0$, we can re-write (17) into the step function

$$\begin{cases} \delta U'' = (\lambda+1)U & 0 \le x \le \ell \\ \delta \tilde{U}'' = (\lambda + c)\tilde{U} & \ell \le x \le \frac{1}{4} \end{cases} \tag{23}$$

where

$$c = 1 - \frac{u_{\max} f'(u_{\max})}{f(u_{\max})}$$

with the **mid-point boundary conditions**

$$U(\ell) = \tilde{U}(\ell) \tag{24a}$$
$$\tilde{U}'(\ell) - \tilde{U}'(\ell) = \Gamma U_*(\ell) \tag{24b}$$

4.2 Solving the mid-point boundary condition

Since $U = u'_{ss}(x)$ is the translational eigenmode for $\lambda = 0$, we first use this solution and (23) to determine Γ in (24b). The piecewise solutions of (23) are given by

$$U(x) = \sinh(\sqrt{\tfrac{1}{\delta}}x) \qquad \tilde{U}(x) = A\sinh(\sqrt{\tfrac{c}{\delta}}(\tfrac{1}{4} - x)),$$

and then (24) takes the form

$$\begin{aligned} \sinh(\sqrt{\tfrac{1}{\delta}}\ell) &= A\sinh(\sqrt{\tfrac{c}{\delta}}\tilde{\ell}) \\ \sqrt{\tfrac{1}{\delta}}\cosh(\sqrt{\tfrac{1}{\delta}}\ell) - \sqrt{\tfrac{c}{\delta}}A\cosh(\sqrt{\tfrac{c}{\delta}}\tilde{\ell}) &= \Gamma\sinh(\sqrt{\tfrac{1}{\delta}}\ell) \end{aligned}$$

yielding

$$\Gamma = \frac{1}{\sqrt{\delta}\tanh(\sqrt{\frac{1}{\delta}}\ell)} - \frac{\sqrt{c}}{\sqrt{\delta}\tanh(\sqrt{\frac{c}{\delta}}\tilde{\ell})}. \tag{25}$$

4.3 Approximating the competition eigenvalue

To satisfy the **competition boundary condition** (18), the solutions $U, \tilde{U}$ are chosen in (23) as

$$U = \sinh(\sqrt{\tfrac{\lambda+1}{\delta}}x) \qquad \tilde{U} = A\cosh(\sqrt{\tfrac{\lambda+c}{\delta}}(\tfrac{1}{4} - x)). \tag{26}$$

Then midpoint boundary condition (24b) can be rewritten with Γ as

$$\begin{aligned} \sinh(\sqrt{\tfrac{\lambda+1}{\delta}}\ell) &= A\cosh(\sqrt{\tfrac{\lambda+c}{\delta}}\tilde{\ell}) \\ \sqrt{\tfrac{\lambda+1}{\delta}}\cosh(\sqrt{\tfrac{\lambda+1}{\delta}}\ell) - \sqrt{\tfrac{\lambda+c}{\delta}}A\sinh(\sqrt{\tfrac{\lambda+c}{\delta}}\tilde{\ell}) &= \Gamma\sinh(\sqrt{\tfrac{\lambda+1}{\delta}}\ell) \end{aligned}$$

This yields the equation

$$\sqrt{\tfrac{\lambda+1}{\delta}}\frac{1}{\tanh(\sqrt{\frac{\lambda+1}{\delta}}\ell)} - \sqrt{\tfrac{\lambda+c}{\delta}}\tanh(\sqrt{\tfrac{\lambda+c}{\delta}}\tilde{\ell}) = \frac{1}{\sqrt{\delta}\tanh(\sqrt{\frac{1}{\delta}}\ell)} - \frac{\sqrt{c}}{\sqrt{\delta}\tanh(\sqrt{\frac{c}{\delta}}\tilde{\ell})}. \tag{27}$$

After rearranging terms and making use of simplifications for small δ, this equation can be reduced to

$$\frac{\lambda}{2} - \frac{\lambda}{2\sqrt{c}} + 2\sqrt{c}e^{-2\tilde{\ell}\sqrt{c/\delta}} \approx 0, \tag{28}$$

which finally yields

$$\lambda_{\text{compete}} \approx A_1 e^{-\sqrt{\frac{c}{\delta}}\ell_{\text{mesa}}} \qquad \text{with} \qquad A_1 = \frac{4c}{1-\sqrt{c}}. \tag{29}$$

4.4 Approximating the merging eigenvalue

Similarly, to satisfy the **merging boundary condition** (19), the solutions $U, \tilde{U}$ are chosen as

$$U = \cosh(\sqrt{\tfrac{\lambda+1}{\delta}}x) \qquad \tilde{U} = A\sinh(\sqrt{\tfrac{\lambda+c}{\delta}}(\tfrac{1}{4} - x)) \tag{30}$$

Substituting these solutions into the midpoint boundary conditions (24b) yields

$$\begin{aligned} \cosh(\sqrt{\tfrac{\lambda+1}{\delta}}\ell) &= A\sinh(\sqrt{\tfrac{\lambda+c}{\delta}}\tilde{\ell}) \\ \sqrt{\tfrac{\lambda+1}{\delta}}\sinh(\sqrt{\tfrac{\lambda+1}{\delta}}\ell) - \sqrt{\tfrac{\lambda+c}{\delta}}A\cosh(\sqrt{\tfrac{\lambda+c}{\delta}}\tilde{\ell}) &= \Gamma\cosh(\sqrt{\tfrac{\lambda+1}{\delta}}\ell) \end{aligned}$$

This system of equations can then be reduced to the condition

$$\sqrt{\tfrac{\lambda+1}{\delta}}\tanh(\sqrt{\tfrac{\lambda+1}{\delta}}\ell) - \frac{\sqrt{\lambda+c}}{\sqrt{\delta}\tanh(\sqrt{\frac{\lambda+c}{\delta}}\tilde{\ell})} = \frac{1}{\sqrt{\delta}\tanh(\sqrt{\frac{1}{\delta}}\ell)} - \frac{\sqrt{c}}{\sqrt{\delta}\tanh(\sqrt{\frac{c}{\delta}}\tilde{\ell})}$$

Again, neglecting smaller terms in the limit that δ is small, we obtain

$$\frac{\lambda}{2} - 2e^{-2\ell/\sqrt{\delta}} - \frac{\lambda}{2\sqrt{c}} = 0$$

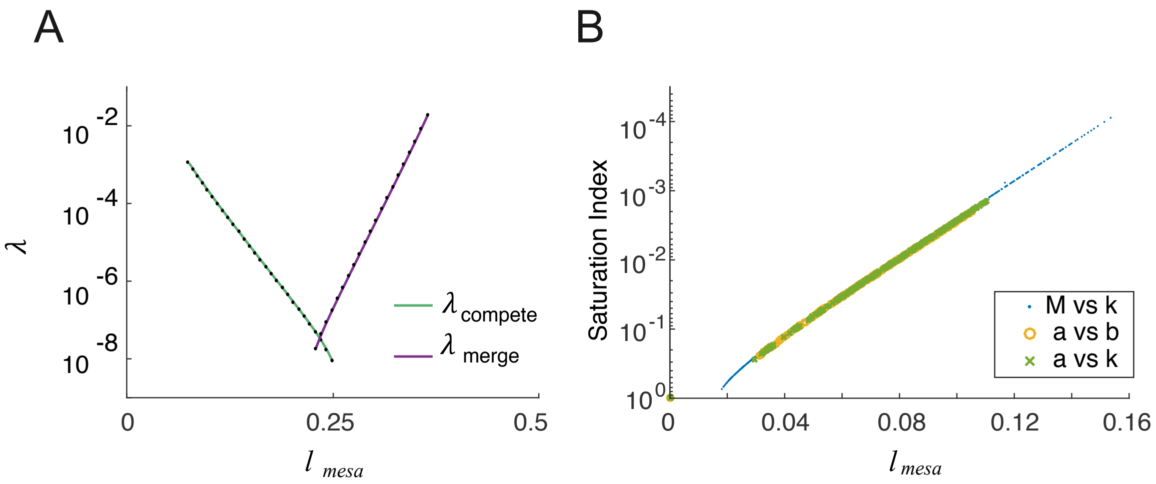

Figure S5: The eigenvalues for competition and merging modes depend exponentially on the saturation index.
(A) The eigenvalues for competition and merging calculated by the shooting method for each steady state solution with varying M from 10 to 32 with an increment of 0.5. (B) Peak width, ℓ_{mesa}, is a robust indicator of the saturation index (defined as $u_{\mathrm{sat}} - u_{\mathrm{max}})/u_{\mathrm{sat}}$) over a broad range of system parameters. Data points collected from simulations in Fig. 4A.

which finally yields

$$\lambda_{\text{merge}} \approx A_2 e^{-\sqrt{\frac{1}{\delta}}\ell_{\text{valley}}} \qquad \text{with} \qquad A_2 = \frac{4\sqrt{c}}{\sqrt{c}-1}, \tag{31}$$

and recall that $\ell_{\text{valley}} = \frac{1}{2} - \ell_{\text{mesa}}$. We can numerically calculate two-peak steady-states over a range of values for ℓ_{mesa} by varying the total mass M. The computed results for λ_{compete} and λ_{merge} for the two-peak steady states confirm these analytical predictions (Fig. S5A).

The width of a mesa is an indicator that perfectly correlates with how close to saturation a peak is. Defining a saturation index as $(u_{\text{sat}} - u_{\max})/u_{\text{sat}}$, and ℓ_{mesa} as normalized by $\sqrt{b/D_u}$, we find that two peak steady states of the dimensional model (eq 5 in the main text) plotted in Fig. 4 in the main text collapse into a perfect correlation, no matter what parameter we change (Fig S5). The relationship between ℓ_{mesa} shows that the wider the mesa, the more saturated the two peak steady states are, and thus the less efficient competition will be.

5 Unifying Turing and Wave-pinning models

As the Turing-type and Wave-pinning models behaved similarly with regard to to competition and saturation, we revisited their behavior with regard to diffusion-driven instability and wave-like spread.

5.1 Wave-pinning models can be Turing unstable

We first explored the behavior of simulations of the Wave-pinning (eq 7) and Turing-type (eq 8) models starting from the homogeneous steady state with random noise for u. In both cases, multiple peaks formed and then rapidly competed. We investigate the stability of the wave-pinning model below, and find that with appropriate parameters, the Wave-pinning model is indeed Turing unstable. (Fig. S6)

The reaction term of the wave-pinning model (7) has three roots. One is the trivial solution, which is always Turing stable:

$$u = 0, \qquad v = M \tag{32}$$

The non-trivial solutions can be obtained from

$$\frac{uv}{1+\kappa u^2} - 1 = 0 \qquad \rightarrow \qquad (\kappa+1)u^2 - Mu + 1 = 0.$$

This yields two solutions

$$u = \frac{M \pm \sqrt{M^2 - 4(\kappa+1)}}{2(\kappa+1)} \qquad v = u - M \tag{33}$$

under the condition

$$M > \sqrt{4(\kappa+1)}.$$

The condition for Turing instability in MCAS models reads as follows [1,2]:

$$\eta F_u - F_v > 0 \tag{34}$$

where $\eta = D_v/D_u$ and F_u and F_v are the derivatives of $F(u,v)$ with respect to u,

$$F_u = \frac{2uv}{(1+\kappa u^2)^2} - 1, \qquad F_v = \frac{u^2}{1+\kappa u^2}.$$

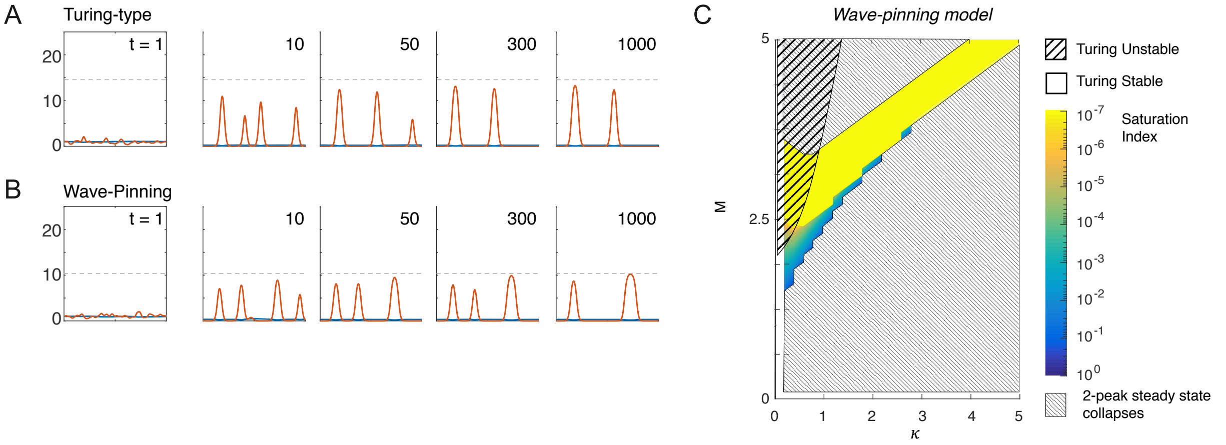

Figure S6: Symmetry breaking and competition of both the Turing-like and the Wave-pinning model. A, B.) Simulations of Turing-type (8) and Wave-Pinning model (7). u is indicated in red, v in blue. Dashed line indicate the saturation point. $M = 2$, $D_u = 0.01$, $D_v = 1$; $\kappa = 0$ for the Turing-type model and $\kappa = 0.01$ for the Wave-Pinning model. C.) The Turing unstable regime is independent of the saturation regime in the wave-pinning model. Stability of the homogeneous steady state was calculated by (35) with parameters $M = 0.005 \sim 5$, $\kappa = 0.01 \sim 5$. Saturation index was extracted from simulated 2-peak steady states with parameters $M = 0.1 \sim 5$, $\kappa = 0.2 \sim 5$. plotted in color in log scale, varying Uncolored regions indicate parameter spaces where the 2-peak steady state collapses to the homogeneous steady state.

Since the homogeneous steady states satisfy

$$\frac{uv}{1+\kappa u^2} - 1 = 0$$

then condition (34) becomes

$$\frac{2\eta - u^2}{1+\kappa u^2} - \eta > 0 \qquad \rightarrow \qquad 2\eta - u^2 > (1+\kappa u^2)\eta.$$

Therefore, the Turing unstable condition in the non-dimensional system (6, 7) reads:

$$u^2\left(\kappa + \frac{1}{\eta}\right) < 1. \tag{35}$$

In the Turing-type model at when k is small, the system is easily Turing unstable due to large ratio η between the two diffusion constants.

5.2 Turing-type models can exhibit wave-pinning behavior.

Wave-pinning dynamics are thought to depend on a bi-stable system [1,3]. As we showed that Turing-type models can exhibit bi-stability due to local depletion of cytoplasmic substrate, they too should be able to manifest wave-pinning dynamics. Indeed, if we start a simulation with one large spike of u and all other material as v, the spike triggers positive feedback and expands in a wave-like manner. As the wave spreads, v is depleted, until eventually the wave-pinning condition eq 14 is satisfied, and the wave stops when the top of the peak corresponds to the saturation point u_{sat} of each model. This behavior is seen in both Turing-type and Wave-pinning models without discernible qualitative differences. (Fig. S7).

In summary, MCAS models may exhibit Turing instability or Wave-pinning dynamics, and may compete effectively or co-exist, depending on parameters. The ”typical” behavior or Turing or Wave-pinning model simply represents behaviors of MCAS models in a specific parameter subspace. This view is consistent with a recent review on MCAS models ([4]).

6 Steady state solutions of 2D MCAS models

In 2-dimensions, the steady state solutions also transition from sharp peaks to mesas as the total mass M is increased for the $D_v \gg D_u$ limit (Fig. S8). With increased M, cytoplasmic v monotonically decreases and approaches a minimal value, indicating that larger peaks possess stronger recruitment power, but recruitment power reaches a limit as peaks saturate. These trends are similar in 1D and 2D, thus the saturation rule can be applied in 2D. However, peak height (u_{max}) of sharp peaks can exceed that of mesas in 2D.

The cause of this discrepancy can be shown by repeating the derivation in **section 2**, but rewriting Eq. 11a in 2-dimensional polar coordinates $u(r,\theta)$.

$$0 = D_u\left(\frac{\partial^2 u}{\partial r^2} + \frac{1}{r}\frac{\partial u}{\partial r} + \frac{1}{r^2}\frac{\partial^2 u}{\partial \theta^2}\right) + F(u,v) \tag{36}$$

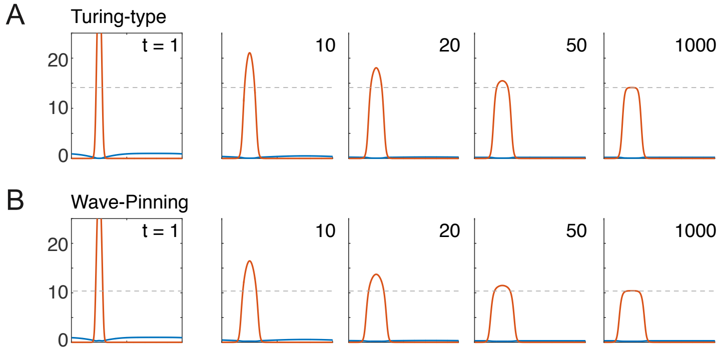

Figure S7: Wave-pinning behavior in both Turing-like (8) and Wave-pinning (7) model. Initial condition for both simulations are $u = 0$, $v = M$, with a spike in u that triggers the wave. u is indicated in red, v in blue. Parameter values are the same as Fig. S6, except that $M = 2.6$.

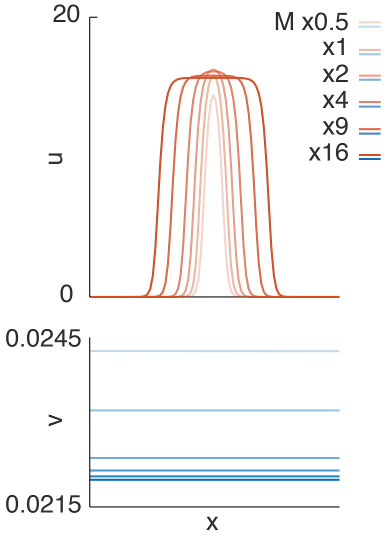

Figure S8: Solutions of the 2-dimensional Wave-pinning model transitions from sharp peaks to mesas. Cross-sections of the steady state solutions along the x-axis. With increased M, v decreases and approaches a lower limit, indicating that peaks with more protein materials possess stronger recruitment power, but the recruitment power reaches a limit as peaks becomes mesas.

For a steady state peak centered at $r = 0$, diffusion sideways on the θ direction is zero, but the $\frac{1}{r}\frac{\partial u}{\partial r}$ term cannot be neglected in the derivation. Therefore, eq 14 does not hold, and the conclusion that there is a largest possible u (u_{sat}) which saturated mesas approach is limited to 1D scenarios.

7 Curvature-driven competition in 2-dimensional space.

Previously developed wave-pinning theory [5–7] indicates that on a 2-dimensional surface, the wave speed c is governed by (see Fig. 8 in the main text):

$$c = c_0(v) - \frac{D_u}{R} \tag{37}$$

When two unequal mesas transiently coexist on a 2-dimensional surface in a $D_v \to \infty$ scenario, each will have its own radius R_1 and R_2 ($R_1 > R_2$), but both mesas will share the same v and thus the same c_0.

$$c_1 = c_0 - \frac{D_u}{R_1} \tag{38a}$$

$$c_2 = c_0 - \frac{D_u}{R_2} \tag{38b}$$

Initially, the wave front of both mesas will spread with positive speeds. As v becomes depleted, c_0 will decrease until it becomes smaller than $\frac{D_u}{R_2}$, and c_2 becomes negative. After this, v halts at a quasi-steady state as the larger mesa expands in expense of the smaller one, and the decrease of the protein content in the smaller mesa (M_2) equals the increase of M_1:

Therefore, in curvature-driven competition, the protein flux from the smaller mesa to the larger

$$\begin{aligned}\frac{dM_1}{dt} = \frac{d}{dt}(\pi R_1^2 h) &= -\frac{dM_2}{dt} = -\frac{d}{dt}(\pi R_2^2 h)\\ \to 2\pi h R_1 \frac{dR_1}{dt} &= -2\pi h R_2 \frac{dR_2}{dt}\\ \to R_1 c_1 &= -R_2 c_2\end{aligned} \tag{39}$$

We can insert (Eq 38) into (Eq 39) and obtain

$$\begin{aligned}R_1 c_0 - D_u &= -R_2 c_0 + D_u\\ \to c_0(R_1 + R_2) &= 2D_u\\ \to c_0 &= \frac{D_u}{\bar{R}}\end{aligned} \tag{40}$$

where $\bar{R}$ is the average of R_1 and R_2.

Therefore, the wave-pinning theory predicts a theoretical value of the protein flux, which we plot in Fig. 9B in the main text:

$$\begin{aligned}\frac{dM_1}{dt} &= 2\pi h R_1 \frac{dR_1}{dt} \\ &= 2\pi h R_1 D_u \left(\frac{1}{\bar{R}} - \frac{1}{R_1} \right)\end{aligned} \tag{41}$$

References

[1] Mori Y, Jilkine A, Edelstein-Keshet L. Wave-pinning and cell polarity from a bistable reaction-diffusion system. Biophys J. 2008;94(9):3684–97. doi:10.1529/biophysj.107.120824.

[2] Rubinstein B, Slaughter BD, Li R. Weakly nonlinear analysis of symmetry breaking in cell polarity models. Phys Biol. 2012;9(4):045006. doi:10.1088/1478-3975/9/4/045006.

[3] Mori Y, Jilkine A, Edelstein-Keshet L. Asymptotic and Bifurcation Analysis of Wave-Pinning in a Reaction-Diffusion Model for Cell Polarization. SIAM J Appl Math. 2011;71(4):1401–1427. doi:10.1137/10079118X.

[4] Goryachev AB, Leda M. Many roads to symmetry breaking: molecular mechanisms and theoretical models of yeast cell polarity. Mol Biol Cell. 2017;28(3):370–380. doi:10.1091/mbc.E16-10-0739.

[5] Tyson JJ, Keener JP. Singular Perturbation-Theory of Traveling Waves in Excitable Media. Physica D-Nonlinear Phenomena. 1988;32(3):327–361. doi:Doi 10.1016/0167-2789(88)90062-0.

[6] Keener JP. A Geometrical-Theory for Spiral Waves in Excitable Media. Siam Journal on Applied Mathematics. 1986;46(6):1039–1056. doi:Doi 10.1137/0146062.

[7] Zykov VS. [Analytic evaluation of the relationship between the speed of a wave of excitation in a two-dimensional excitable medium and the curvature of its front]. Biofizika. 1980;25(5):888–92.
